# Slit2 Protects Hearts Against Ischemia-Reperfusion Injury by Inhibiting Inflammatory Responses and Maintaining Myofilament Contractile Properties

**DOI:** 10.3389/fphys.2020.00228

**Published:** 2020-03-27

**Authors:** Xiang Li, Shuang Zheng, Weijiang Tan, Hongqi Chen, Xiaohui Li, Jian Wu, Ting Luo, Xuecong Ren, W. Glen Pyle, Lijing Wang, Peter H. Backx, Ren Huang, Feng Hua Yang

**Affiliations:** ^1^Guangdong Province Key Laboratory of Laboratory Animals, Cardiovascular Model Research Center, Guangdong Laboratory Animals Monitoring Institute, Guangzhou, China; ^2^School of Basic Medicine, Vascular Biology Institute, Guangdong Pharmaceutical University, Guanghzou, China; ^3^Department of Cardiovascular Surgery, The First Affiliated Hospital, Jinan University, Guangzhou, China; ^4^Shanghai Institute of Cardiovascular Diseases, Zhongshan Hospital and Institutes of Biomedical Sciences, Fudan University, Shanghai, China; ^5^Department of Biomedical Sciences, Ontario Veterinary College, University of Guelph, Guelph, ON, Canada; ^6^Department of Biology, York University, Toronto, ON, Canada; ^7^Division of Cardiology and the Peter Munk Cardiac Centre, University Health Network, Toronto, ON, Canada

**Keywords:** ischemia-reperfusion injury, contractile function, RNA sequencing, myofilament phosphorylation, inflammatory response, cTnI, Slit2, protein kinase C

## Abstract

**Background:**

The secreted glycoprotein Slit2, previously known as an axon guidance cue, has recently been found to protect tissues in pathological conditions; however, it is unknown whether Slit2 functions in cardiac ischemia–reperfusion (IR) injury.

**Methods:**

Langendorff-perfused isolated hearts from Slit2-overexpressing (Slit2-Tg) mice and C57BL/6J mice (background strain) were subjected to 20 min of global ischemia followed by 40 min of reperfusion. We compared Slit2-Tg with C57BL/6J mice in terms of left ventricular function and infarct size of post-IR hearts along with tissue histological and biochemical assessments (mRNA and protein expression, phosphorylation status, and myofilament contractile properties).

**Results:**

Slit2 played cardioprotective roles in maintaining contractile function and reducing infarct size in post-IR hearts. IR increased the expression of the Slit2 receptor Robo4 and the membrane receptor Slamf7, but these increases were suppressed by Slit2 overexpression post IR. This suppression was associated with inhibition of the nuclear translocation of NFκB p65 and reductions in IL-1β and IL-18 release into perfusates. Furthermore, Slit2 overexpression attenuated the increases in myofilament-associated PKCs and phosphorylation of cTnI at Ser43 in the post-IR myocardium. The myofilament calcium sensitivity and actomyosin MgATPase activity were preserved in the post-IR Slit2 myocardium.

**Conclusion:**

Our work demonstrates that Slit2 inhibits inflammatory responses and maintains myofilament contractile properties, thus contributing, at least in part, to the prevention of structural and functional damage during IR.

## Introduction

WHO statistics indicate that the annual deaths from ischemic heart disease were ∼8.9 million worldwide in 2017 (GBD 2017; Causes of Death Collaborators, 2018), despite the availability of various drugs and other therapeutic approaches that have been developed to tackle this epidemic condition (Hausenloy and Yellon, 2013). Reestablishment of blood flow to previously ischemic tissues during treatment results in tissue and functional damage called ischemia–reperfusion (IR) injury (Murphy and Steenbergen, 2008; Prasad et al., 2009). Inflammatory response is the primary mechanism of IR injury, in which reperfusion triggers leukocyte recruitment and reactive oxygen species (ROS) production in endothelial cells. Subsequently, transcriptional factors such as NFκB activate the expression of proinflammatory cytokines TNF-α, IL-1β, IL-6, and IL-18 (Barnes and Karin, 1997; Kawaguchi et al., 2011; Burma et al., 2014; Lu et al., 2016). Blockade of leukocytes’ adherence to endothelial cells significantly reduces the infarct size in the IR myocardium of cats (Ma et al., 1992), and inhibition of NFκB reduces neutrophil activation and cell death in IR rabbit hearts (Yeh et al., 2005).

IR depresses the contractile function of the heart. Cardiac contractile function is executed by myofilaments, including thin filaments [actin, tropomyosin, and the troponin complex of cardiac troponin C (cTnC), cardiac troponin I (cTnI), and cardiac troponin T (cTnT)] and thick filaments [myosin heavy and light chains, myosin binding protein C (MyBP-C), and titin] (van der Velden and Stienen, 2019) and regulated by the balance of myofilament phosphorylation (Metzger and Westfall, 2004; Verduyn et al., 2007; Kooij et al., 2010). Among the phosphorylation sites of myofilaments, Ser23/24 and Ser43/45 of cTnI are the most investigated sites. Ser23/24 and Ser43/45 are preferentially phosphorylated by protein kinase A (PKA) and protein kinase C (PKC) isoforms, respectively (Jideama et al., 1996). Phosphorylation of Ser23/24 accelerates the relaxation rate (Takimoto et al., 2004), and phosphorylation of Ser43/45 reduces maximum ATPase activity (Noland et al., 1995). Protein kinase signaling has been explored as a therapeutic target for improvement of cardiac function in heart disease (Singh et al., 2017).

Slit2 is one of three Slit family members that are secreted from the extracellular matrix and expressed in many tissues, including the hearts (Dickson and Gilestro, 2006). Slit2 can exist in a full-length or in a cleaved form of an NH2-terminal fragment or a COOH-terminal fragment (Nguyen Ba-Charvet et al., 2001). Slit2 is a ligand of the Robo family of immunoglobulin receptors (Robo1–4). The Slit2 isoform, similar to its family members, guides heart tube formation during development (Wong et al., 2002), but the roles of Slit2 in adult hearts are unknown. Evidence has shown that Slit2 protects several organs and tissues through anti-inflammatory mechanisms. For example, Ye et al. (2010) reported that Slit2 suppresses chemotaxis of neutrophils to inhibit lung inflammation in mice. In addition, Zhao et al. (2014) showed that Robo4, a ligand of Slit2, inhibits lipopolysaccharide-induced expression of the cytokine and cell adhesion molecule ICAM-1 in human microvascular endothelial cells. Furthermore, Slit2 inhibits neutrophil and macrophage infiltration in response to IR and reduces renal tubular necrosis in mice (Chaturvedi et al., 2013). As the anti-inflammatory mechanisms of Slit2 that occur during other organ stresses, particularly renal IR, are similar to those that occur during cardiac IR, we hypothesized that Slit2 suppresses inflammatory responses in the IR myocardium.

Slit2 regulates intracellular PKC signaling and plays key roles in development and physiological function. PKC is recruited to colocalize with Robo3 in murine brain regions that govern learning, memory, and reward (Samelson et al., 2015). Increases in Slit2/Robo1 expression caused by prostaglandin F2 are PKC-dependent during murine luteolysis (Zhang et al., 2013). In addition, PKC-dependent substrate phosphorylation is required for F-actin function in murine commissural growth cones in response to Slit2 (Shen et al., 2002). However, under pathological conditions, Slit2-associated PKC activation might exert detrimental effects. For example, treatment with phospholipase C (PLC), the upstream protein of PKC (Essen et al., 1997), impairs Slit2-induced angiogenesis in cultured endothelial cells (Dunaway et al., 2011). In the myocardium, PKC includes more than 10 isoenzymes that might play beneficial or detrimental roles upon activation (Steinberg, 2012). For example, PKCδ contributes to ATP depletion, ROS accumulation, apoptosis, and infarction during cardiac IR (Inagaki et al., 2003a; Murriel et al., 2004), while treatment with a PKCδ inhibitor reduces infarct size and improves the functions of reperfused swine hearts (Inagaki et al., 2003a). Additionally, PKCε, another intensively investigated isoform, shows either protective or undetectable effects during the reperfusion period (Inagaki et al., 2003b). These results suggest that Slit2 might regulate PKC-dependent contractile function during IR.

In this study, we aimed to explore the effects of Slit2 and its regulatory mechanisms regarding anti-inflammatory responses and myofilament phosphorylation in the post-IR myocardium. Understanding the regulatory mechanisms of Slit2 will provide a novel therapeutic strategy for combating IR injury.

## Materials and Methods

### Animals

C57BL/6 mice overexpressing the Slit2 gene (Slit2-Tg) were originally generated by the Geng Laboratory (University of Michigan, United States) (Yang et al., 2010). Briefly, full-length human Slit2 cDNA was cloned into a pCEP4F vector containing the CMV promoter, and then injected into the pronuclei of fertilized C57 × CBA F1 oocytes to construct transgenic mice. The genotypes were confirmed by Southern blotting and PCR analysis. PCR screening of Slit2 heterozygotes was performed using a pair of primers specific for Slit2 cDNA (forward: 5′-CCCTCCGGATC CTTTACCTGTCAAGGTCCT-3′; reverse: 5′-TGGAGAGAGCTCACAGAACAAGCCACTGTA-3′). Slit2-Tg mice and C57BL/6J (background strain) mice were bred and maintained in a specific pathogen-free (SPF), AAALAC-accredited facility of the Guangdong Laboratory Animals Monitoring Institute, Guangzhou, China. The temperature and humidity were 24 ± 2°C and 40–60%, respectively, and the light cycle included 12 h of light and 12 h of dark. Three- to five-month-old male mice were used in this study. All animal experiments were approved by the Institutional Animal Care and Use Committee (No. IACUC2017002).

### Langendorff Heart Preparation and Perfusion Protocol

To determine the effects of Slit2 on myocardial IR injury, a Langendorff heart perfusion system for rodents (Radnoti LLC, United States) equipped with a pressure transducer was used (Yang and Pyle, 2012). Briefly, mice were euthanized with isoflurane, and their hearts were excised. The aortas were then cannulated and the hearts were perfused using a modified Krebs-Henseleit (KH) buffer solution (37°C) containing (in mM) 118 NaCl, 4.7 KCl, 1.2 MgSO_4_, 2.5 CaCl_2_, 1.2 KH_2_PO_4_, 25 NaHCO_3_, 0.05 EDTA, 0.5 sodium pyruvate, and 11 glucose. The KH buffer was continuously gassed with 95% O_2_/5% CO_2_, and the perfusion pressure was maintained at 70 mmHg. A water-filled balloon connected to the pressure transducer was inserted into the left ventricle, and left ventricular pressures were recorded with a PowerLab 16/35 system (AD Instruments Inc., United States). Hemodynamic parameters, including the left ventricular developed pressure (LVDP), heart rate (HR), left ventricular end diastolic pressure (LVEDP), rate of pressure product (RPP), and minimum and maximum rates of left ventricular pressure development [dP/dt(min) and dP/dt(max), respectively] were analyzed with Chart 7 Software (ADInstruments Inc., United States).

**FIGURE 1:**
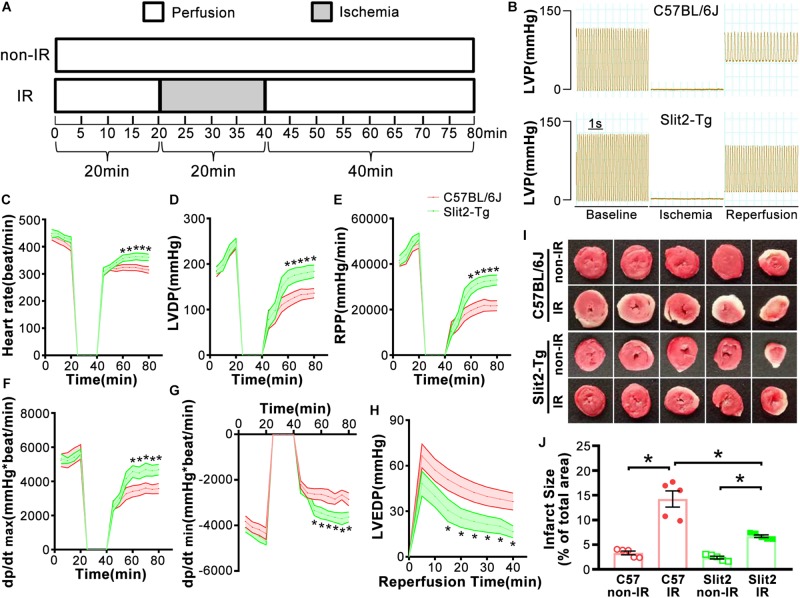
Effects of Slit2 on post-ischemia–reperfusion (IR) cardiac function. **(A)** Perfusion protocol. All hearts were perfused for 20 min for equilibration. The IR groups were subjected to 20 min of global ischemia and 40 min of reperfusion, while the non-IR groups were perfused continuously for 80 min without global ischemia. **(B)** Representative traces of left ventricular pressure (LVP) for C57BL/6J (top) and Slit2-Tg (bottom) hearts during IR. Functional measurements of C57BL/6J and Slit2-Tg hearts were taken. **(C–H)** Heart rate, left ventricular developed pressure (LVDP), rate pressure product (RPP), dP/dt(max), dP/dt(min), and left ventricular end-diastolic pressure (LVEDP) values. *n* = 7 mice per group. The values are the means ± SEMs; *P < 0.05 vs. C57BL/6J hearts (unpaired Student’s *t*-test). **(I)** Representative images of serial TTC-stained sections of C57BL/6J and Slit2-Tg hearts subjected or not subjected to IR. **(J)** Quantitative analysis of the infarct area in each group. *n* = 5 mice per group. All data are presented as the mean ± SEM; *P < 0.05 between the groups (two-way ANOVA, Tukey’s multiple comparisons test).

For the IR protocol, hearts were subjected to 20 min of stable perfusion, followed by 20 min of no-flow global ischemia and 40 min of reperfusion. Control hearts were continuously perfused for 80 min (Figure 1A). At the end of the experiment, some heart tissues were fixed for hematoxylin–eosin (HE) staining, transmission electron microscopy (TEM), and immunofluorescence staining, and some were flash frozen in liquid nitrogen for subsequent RNA sequencing analysis, RT-qPCR, Western blotting, Pro-Q staining, and actomyosin MgATPase activity assay.

### Myocardial Infarct Size

To determine post-IR myocardial infarct size, hearts were subjected to TTC staining. Briefly, the frozen tissues were sliced into 2 mm thick sections and incubated in a 1% TTC (Sigma-Aldrich, United States) solution at 37°C for 15 min and in 10% neutral formalin for 1 h. TTC stained the viable areas red, while the unstained areas (white) were infarcted tissue. The infarct size in the myocardial tissue was measured using ImageJ software (NIH, United States), and the infarct size (%) was calculated as a percentage of the total section area. Different technicians performed the sectioning and observations, and the technicians did not know the individual identities of the samples.

### HE Staining

Hearts were fixed in 10% neutral formalin and subjected to HE staining, which was used to detect necrosis of heart tissues. First, the hearts were embedded in paraffin, sliced into 3 μm sections, and dewaxed with dimethylbenzene and dehydrated with gradient ethanol solutions. Next, the sections were stained with hematoxylin for 10 min and soaked in 1% hydrochloric acid–ethanol for 2 s. The sections were then stained with alcohol-soluble eosin for 25 s. Finally, the sections were sealed with neutral gum and observed under an optical microscope (Leica DM 2000, Germany). During evaluation of the histological changes, different technicians performed the sectioning and observations, and the technicians did not know the sample IDs.

### Transmission Electron Microscopy

TEM was further used to determine the subcellular changes associated with Slit2 in the post-IR myocardium. Left ventricular tissues were cut into small blocks (about 1 mm^3^), fixed with 2.5% glutaraldehyde, fixed with 1% OsO_4_, dehydrated in ethanol, and embedded in Araldite. The tissue blocks were cut into slices at a thickness of 60 nm using a Leica cryostat system (EM UC7/FC7, Germany) and collected on copper grids. The ultrathin sections were double stained with 3% uranyl acetate and lead citrate. The subcellular structure was observed with a Tecnai G2 Spirit transmission electron microscope (FEI Company, United States). During evaluation of the subcellular differences between the groups, different technicians performed the slice preparation and observations, and the technicians did not know the sample IDs.

### RNA Sequencing and Raw Data Processing

Total RNA was isolated using TRIzol^®^ Reagent (Thermo Fisher Scientific, United States), and RNA concentrations were determined using a Qubit^®^ 2.0 Fluorometer and an assay kit (Life Technologies, United States). RNA integrity was assessed using an RNA Nano 6000 Assay Kit with a Bioanalyzer 2100 system (Agilent Technologies, United States). Sequencing libraries were generated using an NEBNext^®^ Ultra^TM^ RNA Library Prep Kit for Illumina^®^ (NEB, United States). After adding index codes, the samples were clustered with a cBot Cluster Generation System using a TruSeq PE Cluster Kit v3-cBot-HS (Illumina, United States). An Illumina HiSeq platform (Illumina, United States) was employed to sequence the libraries, generating 125 bp/150 bp paired-end reads. Raw data with proper paired-end clean reads were mapped for further analysis. The original reads of the genes for the samples in the four groups (the C57-non-IR, C57-IR, Slit2-non-IR, and Slit2-IR groups) were compared in pairs, and the fold changes in the expressed genes between the groups were log2 transformed. A *P* value < 0.05 was the cutoff for identification of differentially expressed genes (DEGs). Furthermore, DEGs with log2 (fold change) values > 0 were considered upregulated, while those with log2 (fold change) values < 0 were considered downregulated. Thus, we defined eight datasets of DEGs: an upregulated dataset and a downregulated dataset each for the C57-IR vs. C57-non-IR, Slit2-IR vs. Slit2-non-IR, Slit2-non-IR vs. C57-non-IR, and Slit2-IR vs. C57-IR comparisons. The gene expression profiles are presented using volcano plots (Supplementary Figure S3). The raw RNA-seq data have been deposited in NCBI’s Gene Expression Omnibus (GEO) (#GSE133796)^1^.

### Target Gene Prediction and Gene Set Enrichment Analysis (GSEA)

First, to identify genes regulated by Slit2 in post-IR hearts, five datasets of DEGs (the downregulated DEGs in the C57-IR vs. C57-non-IR comparison, the upregulated DEGs in the C57-IR vs. C57-non-IR upregulated, the downregulated DEGs in the Slit2-IR vs. C57-IR comparison, the upregulated DEGs in the Slit2-IR vs. C57-IR comparison, and the up-/downregulated DEGs in the Slit2-non-IR vs. C57-non-IR comparison) were input into the TBtools program^2^ to generate a Venn diagram. By analyzing this diagram, two final intersections were obtained: (1) the intersection of the upregulated DEGs in the C57-non-IR vs. C57-IR comparison and the downregulated DEGs in the Slit2-IR vs. C57-IR comparison, and (2) the intersection of the downregulated DEGs in the C57-IR vs. C57-non-IR comparison and the upregulated DEGs in the Slit2-IR vs. C57-IR comparison. These two final intersections further excluded the up-/downregulated DEGs in theSlit2-non-IR vs. C57-non-IR comparison. We therefore identified two new sets of DEGs: one set was upregulated by IR in C57BL/6J hearts but downregulated by Slit2 overexpression in post-IR hearts, and another was downregulated by IR in C57BL/6J hearts but upregulated by Slit2 overexpression in post-IR hearts. The Venn diagram results are presented in Figures 4B,C.

Second, to predict the effects of Slit2 on cardiac function, the GSEA tool Metascape (Zhou et al., 2019) was used to construct protein interaction networks of gene sets. Briefly, the enrichment gene sets (Biological Processes, KEGG pathways, or Reactome pathways) with similar functions were clustered, and the identification algorithm “MCODE” revealed that these enrichment clusters formed interaction networks. The interaction network of the Slit2-non-IR vs. C57-non-IR suggested the effects of Slit2 on the molecular function of the heart (Figures 3A,B), and the interaction network of the Slit2-IR vs. C57-IR suggested the effects of Slit2 on molecular function in post-IR hearts (Figures 3C,D).

### RT-qPCR

RT-qPCR was used to verify the expression of genes identified by the Venn diagram analysis and to determine the expression of Robo receptors. Total RNA extracted from the left ventricle was subjected to reverse transcription and quantitative PCR. The primers are listed in Supplementary Table S1). TB Green^®^ Premix Ex Taq^TM^ II (Takara Bio Inc., Japan) was used. The program was as follows: 95°C for 30 s (1 cycle); 95°C for 5 s, 60°C for 34 s (40 cycles); and 72°C for 10 min (1 cycle). Gene expression levels were normalized to GAPDH levels, and the results were expressed as the ratio of the expression in each experimental group to that in the C57-non-IR group.

### Immunofluorescence Staining

Immunofluorescence detection of Slamf7 and nuclear translocation of NFκB were used to assess inflammatory responses. Heart tissues were fixed in 10% neutral formalin, cryoprotected in 30% sucrose for 24 h, embedded in optimal cutting temperature (OCT) compound, and sectioned with a freezing microtome at a thickness of 5 μm (Leica CM1950, Germany). The heart sections were processed using a standard immunostaining protocol. After routine hydration, the sections were subjected to permeabilization with PBS containing 0.05% Triton X-100 followed by incubation with a primary antibody against Slamf7 (A5782, ABclonal, China) or NFκB p65 (8242S, CST, United States) at 4°C overnight. The primary antibodies were visualized using Alexa Fluor 594- and Alexa Fluor 488-conjugated secondary antibodies (Invitrogen, CA). The sections were photographed under a confocal microscope (Leica TCS SP5, Germany). The technicians performing immunofluorescent staining and observations were different and did not know the sample ID.

### Enzyme-Linked Immunosorbent Assay (ELISA)

Heart perfusates were collected at the perfusion baseline and 0, 5, 10, and 40 min after reperfusion. ELISA kits (Cusabio Biotech Co., China) were used to test the levels of the proinflammatory cytokines IL-1β and IL-18 in the perfusates according to the manufacturer’s instructions. Briefly, 100 μl of the standard or sample was added to each well, and the plates were incubated for 2 h at 37°C. After removing the liquid, 100 μl/well of anti-biotin antibody was added, and the plates were incubated for 1 h at 37°C before being incubated with 100 μl of HRP-avidin and 90 μl of TMB substrate for 25 min at 37°C in the dark. The optical density was measured within 5 min using a microplate reader at 450 nm.

### Myofilament Isolation for Western Blotting, Myofilament Phosphorylation Analysis, and Actomyosin MgATPase Activity Assay

Myofilaments were extracted according to a modified protocol from Yang et al. (2008) and Yang and Pyle (2011, 2012). Briefly, hearts were homogenized in ice-cold lysis buffer composed of (in mM) 60 KCl, 30 imidazole (pH 7.0), 2 MgCl_2_, 0.01 leupeptin, 0.1 phenylmethylsulfonyl fluoride (PMSF), and 0.2 benzamidine and then centrifuged at 12,000 × *g* and 4°C for 15 min. The pellets were extracted with ice-cold lysis buffer containing 1% Triton X-100 and centrifuged at 1,100 × *g* to obtain myofilaments. The protein concentrations of the myofilaments were determined with a bicinchoninic acid (BCA) protein assay kit (Pierce, United States).

### Western Blotting

First, cytosolic fractions extracted from cardiac tissues were used to detect the expression of protein-encoding genes selected from RNA-seq analyses, Slit2 and the Slit2 fragment. Briefly, hearts were homogenized with a cell lysis buffer (#9803, CST, United States) containing protease inhibitors and 1% Triton X-100. The samples were centrifuged at 12,000 × *g* and 4°C for 15 min, and the supernatants were retained for use. Primary antibodies against Slamf7 (A5782, ABclonal, China), RasGRP1 (sc-365358, Santa Cruz, United States), Slit2 (ab7665, Abcam, United States), and Slit2-C (BM5312, Bostern, China) were used. Second, myofilament fractions extracted from cardiac tissues were used to determine myofilament-associated PKCs, PKA, protein phosphatases (PPs), and phosphorylation sites of cardiac myofilaments. Primary antibodies against PKCδ (2058S, CST, United States), PKCε (2683S, CST, United States), PKA (4782S, CST, United States), PP1α (2582S, CST, United States), PP type 2A (PP2A; 2038S, CST, United States), p-cTnISer43 (PA5-35412, Invitrogen, CA), p-cTnISer23/24 (4004S, CST, United States), Slit2 (ab7665, Abcam, United States), Slit2-C (BM5312, Bostern, China), GAPDH (2118S, CST, United States), and actin (MAB1501R, Millipore, United States) were used.

Cytosolic (40 μg) or myofilament (60 μg) proteins were separated using 10% SDS-PAGE and transferred to PVDF blotting membranes (Millipore, United States). After blocking with 5% skim milk, the membranes were incubated with primary antibodies (as listed above) overnight at 4°C before being incubated with species-appropriate secondary antibodies (CST, United States). The bands were detected with Immobilon Western chemiluminescent HRP substrate (Millipore, United States). The densities of the bands were analyzed using ImageJ software (NIH, United States).

### Myofilament Phosphorylation

The phosphorylation levels of myofilament proteins (MyBP-C, desmin, cTnT, and cTnI) were measured using the Pro-Q Diamond phosphorylation gel staining method, as previously described (Yang and Pyle, 2011, 2012). Briefly, myofilaments (60 μg) were separated using 12% SDS-PAGE. After fixation with 50% methanol/10% acetic acid for 30 min, the gels were incubated with Pro-Q Diamond phosphorylation gel staining solution (Molecular Probes, United States) for 90 min. The phosphorylated bands were visualized using a Gel Doc^TM^ XR + gel documentation system (Bio-Rad Laboratories Ltd., United States), and the band densities were quantified using ImageJ (NIH, United States). Total protein was stained with Coomassie solution, and actin bands were used as the loading controls.

### Actomyosin MgATPase Activity

An actomyosin MgATPase activity assay was performed as described previously (Yang et al., 2008; Yang and Pyle, 2011, 2012) to assess myofilament function. Briefly, myofilaments (50 μg) were incubated in reaction solutions containing various concentrations of free calcium. The free calcium concentration in each solution was calculated using a Patton assay program (Patton et al., 2004). The myofilaments were incubated in the reaction solutions for 5 min at 32°C. After the reactions were stopped with 10% trichloroacetic acid, the amount of inorganic phosphate produced was measured after adding 0.5% FeSO_4_ and 0.5% ammonium molybdate in 0.5 M H_2_SO_4_ at 630 nm using a spectrophotometer (Biochrom, United Kingdom).

### Statistical Analysis

All experimental data are presented as the mean ± SEM. Statistical differences between two groups were analyzed with unpaired Student’s *t*-tests, and differences among multiple groups of data were analyzed by two-way ANOVA followed by Tukey’s multiple comparisons test (GraphPad Prism 7, United States). *P* < 0.05 were considered to indicate statistical significance.

## Results

### Slit2 Ameliorates Myocardial Decline and Prevents Cardiac Damage in the Post-IR Myocardium

**FIGURE 2:**
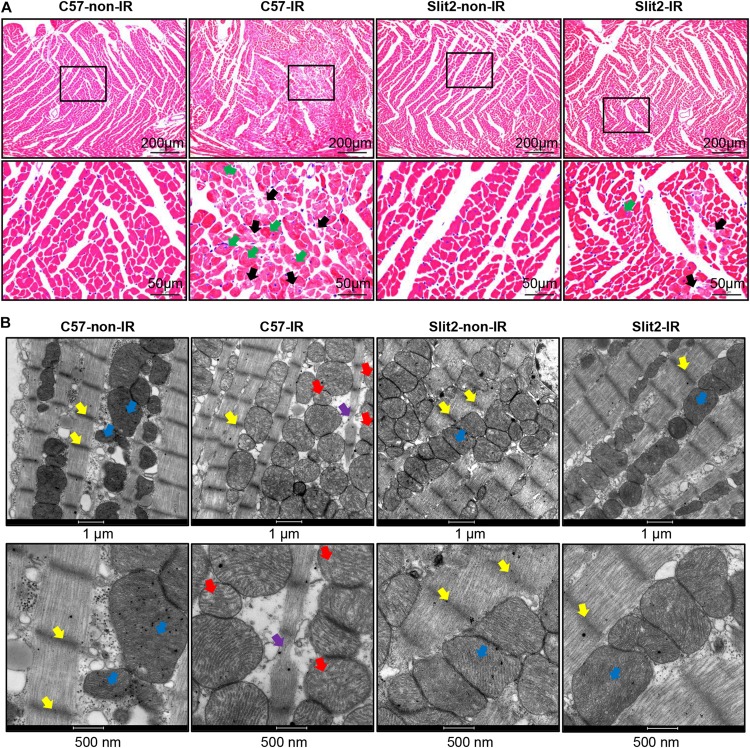
Post-ischemia–reperfusion (IR) injury in C57BL/6J and Slit2-Tg hearts. **(A)** Representative images of hematoxylin–eosin (HE) staining in C57BL/6J and Slit2-Tg hearts. Scale bar = 200 μm (top) or 50 μm (bottom). The bottom panels show the magnified views of the black framed areas of the corresponding upper panels. The black framed area of the C57-IR heart section displays necrosis. The black arrows indicate ruptured cardiomyocytes; the green arrows indicate swollen cardiomyocytes. **(B)** Subcellular structures of cardiomyocytes as examined by TEM in C57BL/6J and Slit2-Tg hearts. Scale bar = 1 μm (top) or 500 nm (bottom). C57-non-IR, C57BL/6J hearts not subjected to IR; Slit2-non-IR, Slit2-Tg hearts not subjected to IR; C57-IR, C57BL/6J hearts subjected to IR; Slit2-IR, Slit2-Tg hearts subjected to IR. Arrows: yellow, Z-discs; blue, normal mitochondria; red, post-IR mitochondria with disappearing crista; purple, ruptured Z-discs.

First, we examined cardiac function in all groups. There were no significant differences in cardiac function (HR, LVDP, and RPP) between C57BL/6J and Slit2-Tg mice at baseline (0–20 min, Figures 1C–E). However, following 20 min of ischemia, HR, LVDP, RPP, and dP/dt(max) were significantly higher and dP/dt(min) was significantly lower in Slit2-Tg mice than in C57BL/6J mice at the time points of 60, 65, 70, 75, and 80 min (Figures 1C–H). These improvements in contractility were associated with lower LVEDP values at the above time points in post-IR Slit2-Tg hearts compared to control hearts (Figure 1H, Table 1, and Supplementary Figure S2) as well as with markedly reduced infarct size in post-IR Slit2-Tg hearts (Figures 1I,J). These results establish that Slit2 protein overexpression significantly improves cardiac systolic and diastolic function following IR and reduces infarct area. The functional parameters are presented in Table 1. Next, we explored the effects of Slit2 on cardiac cellular damage post IR. Under a microscope, we found focal necrosis, some vacuoles, and swollen and dissolved myofibers in cardiac tissues of post-IR C57BL/6J mice (Figure 2A). However, Slit2 overexpression reduced the IR-induced myofiber swelling and vacuole formation between fibers seen in C57BL/6J hearts (Figure 2A). We further employed TEM technology to examine the subcellular structure in post-IR hearts. We found that reperfused C57BL/6J hearts displayed edematous mitochondria, fractured internal crests of mitochondria, ruptured myofibrils, and ruptured Z-discs in cardiomyocytes (Figure 2B). Compared with C57BL/6J hearts, Slit2-Tg hearts had less mitochondrial edema and better myofilament maintenance and Z-line morphology (Figure 2B) after reperfusion. The results from HE staining and TEM examination demonstrate that Slit2 reduces tissue and cellular damage in post-IR hearts.

**TABLE 1:** Hemodynamic analysis of Slit2-Tg and C57BL/6J mouse hearts after IR.

	**HR (beats/min)**	**LVDP (mmHg)**	**dP/dt(max) (mmHg/s)**	RPP (mmHg*beats/min)	**LVEDP (mmHg)**
C57-non-IR	336.413.7	122.711.87	5,821326	40,4473,004	0.6100.60
Slit2-non-IR	360.211.1	119.04.20	5,490303	41,5182,067	1.1840.86
C57-IR	314.312.4	68.025.08*	3,574195*	21,6802,235*	36.425.57*
Slit2-IR	361.111.7^#^	92.016.88*^#^	4,663305*^#^	32,8852,115*^#^	16.503.91*^#^

n = 7. All data are presented as the mean ± SEM. *P < 0.05 vs. non-IR and ^#^P < 0.05 vs. C57-IR (two-way ANOVA, Tukey’s multiple comparisons test). HR, heart rate; LVDP, left ventricular developed pressure; dp/dt(max), maximum increase rate of LV pressure; RPP, rate pressure product; LVEDP, left ventricular end-diastolic pressure.

### Slit2 Shifts Gene Expression Profiles to Boost the Regulation of Cardiac Contraction and Reduce Inflammation in Normal and Post-IR Slit2 Hearts

**FIGURE 3:**
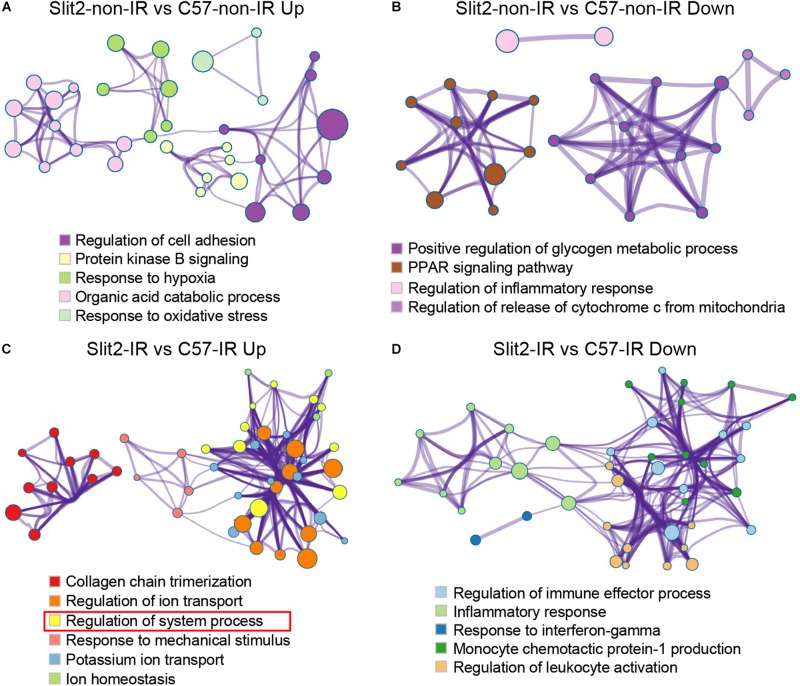
Gene set enrichment analysis of differentially expressed genes (DEGs). Shown are the results for the upregulated DEGs in the Slit2-non-IR vs. C57-non-IR comparison **(A)**, the downregulated DEGs in the Slit2-non-IR vs. C57-non-IR comparison **(B)**, the upregulated DEGs in the Slit2-IR vs. C57-IR comparison (the rectangles in red indicate gene sets enriched for muscle contraction, regulation of heart rate, regulation of heart contraction, heart contraction, actin-mediated cell contraction, regulation of blood circulation, and adrenergic signaling in cardiomyocytes) **(C)**, and the downregulated DEGs in the Slit2-IR vs. C57-IR comparison **(D)**. In the Metascape protein interaction network, two or more gene sets enriched for biological processes/KEGG pathways/Reactome pathways with similar functions were clustered. Within each cluster, the gene set with the highest enrichment score (log *P-*value) was used to annotate the cluster. The links between the clusters are shown. The cluster annotations are color-coded. The size of a node (representing an enriched gene set) is proportional to the number of DEGs in the set, and the width of an edge is proportional to the number of genes shared between the two enriched sets.

Slit2 mRNA and protein levels were significantly higher in Slit2-Tg hearts than in C57BL/6J (background strain) hearts, but there were no functional or histological differences (Supplementary Figure S1 and Supplementary Table S2). The overall gene expression profiles are displayed in Supplementary Figure S3 and Supplementary Table S4. Furthermore, the RNA sequencing data revealed that multiple molecular functions were altered in Slit2-Tg hearts (Figure 3). Slit2 overexpression boosted regulation of cell adhesion, protein kinase B (Akt) signaling, the response to hypoxia, organic acid catabolic processes, and the response to oxidative stress (Figure 3A and Supplementary Table S3). Slit2 reduced positive regulation of glycogen metabolic processes, the PPAR signaling pathway, regulation of the inflammatory response, and regulation of the release of cytochrome C from mitochondria (Figure 3B and Supplementary Table S3). These results suggest that hearts overexpressing Slit2 are sensitive to external stimuli, while they resist inflammatory insults and might exhibit improved cell survival.

In hearts with post-IR injury, Slit2 enhanced collagen chain trimerization, regulation of ion transport, regulation of system processes, responses to mechanical stimuli, potassium ion transport, and ion homeostasis (Figure 3C and Supplementary Table S3). Moreover, Slit2 specifically protected cardiac contractile function in the post-IR myocardium, as observed from the enrichment of the “regulation of system process” term: a cluster of gene sets upregulated in post-IR Slit2 hearts was enriched for muscle contraction, regulation of HR, regulation of heart contraction, heart contraction, actin-mediated cell contraction, regulation of blood circulation, and adrenergic signaling in cardiomyocytes (Figure 3C and Supplementary Table S3). In addition, Slit2 reduced regulation of immune effector processes, the inflammatory response, the response to interferon-gamma, monocyte chemotactic protein-1 production, and regulation of leukocyte activation in the post-IR myocardium (Figure 3D and Supplementary Table S3). These results suggest that Slit2 maintains cardiac contractile function and extracellular structure and prevents inflammatory responses during IR.

### Slit2 Regulates Robo1, Robo4, and Slamf7 and Inhibits Inflammatory Responses in the Post-IR Myocardium

**FIGURE 4:**
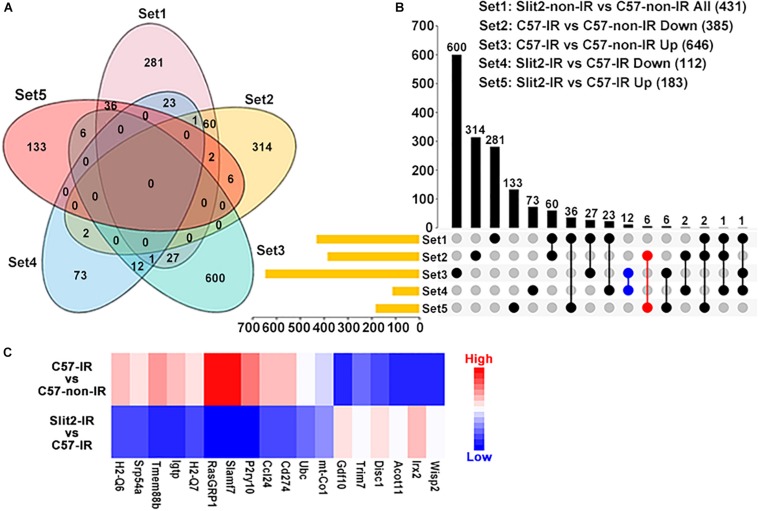
Gene expression profiles of C57BL/6J and Slit2-Tg hearts subjected or not subjected to ischemia–reperfusion (IR) (*n* = 3 per group), as determined by RNA sequencing analysis. **(A)** Venn diagram of the differentially expressed genes (DEGs) in all comparisons, including all DEGs in the Slit2-non-IR vs. C57-non-IR comparison (pink), the upregulated DEGs in the C57-IR vs. C57-non-IR comparison (green), the downregulated DEGs in the C57-IR vs. C57-non-IR comparison (orange), the upregulated DEGs in the Slit2-IR vs. C57-IR comparison (red), and the downregulated DEGs in the Slit2-IR vs. C57-IR comparison (blue). **(B)** UpSet plot displaying the number of DEGs between groups for each intersection. The blue dots represent the genes (12) at the intersection between the downregulated DEGs in the Slit2-IR vs. C57-IR comparison and the upregulated DEGs in the C57-IR vs. C57-non-IR comparison, and the red dots represent the genes (6) at the intersection between the upregulated DEGs in the Slit2-IR vs. C57-IR comparison and the downregulated DEGs in the C57-IR vs. C57-non-IR comparison; both exclude overlap with all DEGs in the Slit2-non-IR vs. C57-non-IR comparison. **(C)** Heatmap displaying the fold changes in 18 genes obtained from the Venn diagram analysis. DEGs with log2 (fold change) values > 0 are indicated in red, and those with log2 (fold change) values < 0 are indicated in blue; a darker color indicates a higher *P-*value.

In exploring the intracellular targets of Slit2 in the protected myocardium, we identified 385, 646, 112, 183, and 431 DEGs from among the downregulated DEGs in the C57-IR vs. C57-non-IR comparison, the upregulated DEGs in the C57-IR vs. C57-non-IR comparison, the downregulated DEGs in the Slit2-IR vs. C57-IR comparison, the upregulated DEGs in the Slit2-IR vs. C57-IR comparison, and the up-/downregulated DEGs in the Slit2-non-IR vs. C57-non-IR comparison, respectively (Figures 4A,B and Supplementary Table S4). By using Venn diagram analysis (Figures 4A,B), we identified 18 genes (6 upregulated and 12 downregulated) that might be regulated by Slit2 to protect the post-IR myocardium. The genes are shown in Figure 4C.

**FIGURE 5:**
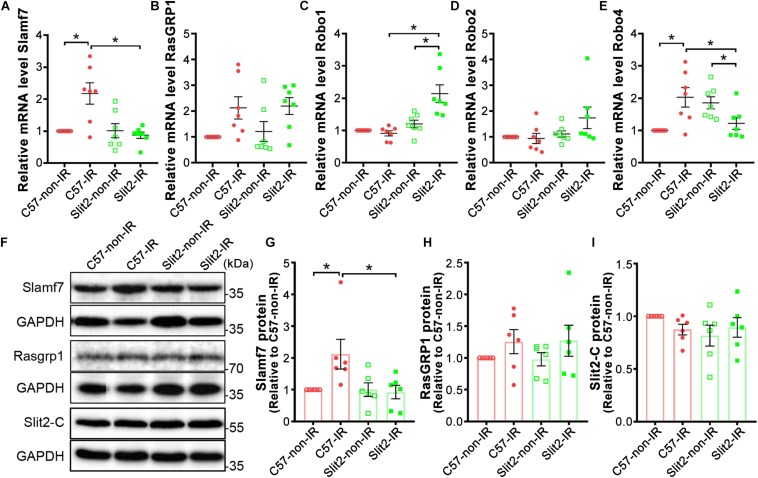
Expression of Slamf7, RasGRP1, Robos, and the Slit2-C fragment in C57BL/6J and Slit2-Tg hearts. **(A–E)** Gene expression of Slamf7 **(A)**, RasGRP1 **(B)**, Robo1 **(C)**, Robo2 **(D)**, and Robo4 **(E)**. *n* = 7 mice per group. **(F)** Representative Western blots of Slamf7, RasGRP1, Slit2-C, and GAPDH (the loading control). **(G–I)** Quantitative results for Slamf7 **(G)**, RasGRP1 **(H)**, and the Slit2-C fragment **(I)** normalized to GAPDH. The values are relative to those in the C57-non-IR group. *n* = 6 mice per group. All data are presented as the mean ± SEM; *P < 0.05 between the groups (two-way ANOVA, Tukey’s multiple comparisons test).

The expression of the two genes most affected by Slit2 overexpression post IR (Slamf7 and RasGRP1) was verified using RT-qPCR and Western blotting. The gene expression and protein expression of Slamf7 were both upregulated by IR in C57BL/6J hearts, but Slit2 overexpression blunted this upregulation in post-IR conditions (Figures 5A,F,G). No differences in RasGRP1 were detected between the groups (Figures 5B,F,H). In addition, RT-qPCR and Western blotting were used to determine the gene and/or protein expression of the Slit2-C fragment and Robo receptors. The protein expression of Slit2-C did not differ between the groups (Figure 5I). Robo1 expression did not change in C57BL/6J hearts after IR but was activated in post-IR Slit2-Tg hearts (Figure 5C). Robo2 was not different between any groups (Figure 5D). Robo4 was upregulated by IR in C57BL/6J hearts, but the IR-induced increases were blunted in Slit2-Tg hearts (Figure 5E). Therefore, we identified Robo1, Robo4, and Slamf7 to be regulated by Slit2 in post-IR hearts.

**FIGURE 6:**
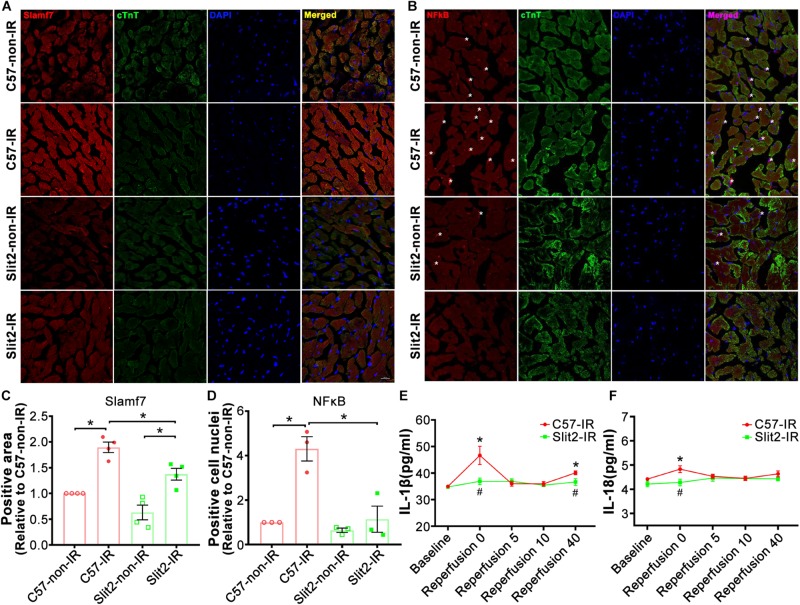
Immunofluorescence staining of Slamf7 and NFκB in the myocardium. **(A–D)** Representative confocal images of Slamf7 (red) **(A)** and NFκB p65 (red) **(B)** in the left ventricular myocardium in C57BL/6J and Slit2-Tg mice. Images of cTnT staining (green), nuclear staining (blue), and merged staining are displayed. Scale bar = 100 nm. The relative levels of Slamf7 were calculated by determining the Slamf7 + area as a percentage of the cTnT + area **(C)**, and the relative levels of NFκB p65 were calculated by determining the number of NFκB p65 + DAPI + cell nuclei (asterisk) as a percentage of the number of DAPI + cell nuclei **(D)**. *n* = 3–4 mice per group. All data are presented as the mean ± SEM; *P < 0.05 between the groups (two-way ANOVA, Tukey’s multiple comparisons test). **(E,F)** Release of the inflammatory cytokines IL-1β **(E)** and IL-18 **(F)** at different time points of reperfusion. *n* = 5 mice per group. All data are presented as the mean ± SEM; *P < 0.05 vs. baseline; ^#^*P* < 0.05 vs. reperfusion at 0 min (unpaired Student’s *t*-test).

A previous study has shown that Slit-Robo signaling plays anti-inflammatory roles, and our RNA sequencing data indicated that Slit2 overexpression reduced inflammatory responses in post-IR conditions (Figure 3D and Supplementary Table S3). In addition, RNA sequencing and expression verification confirmed that Slamf7 was an effector of Slit2 overexpression. Slamf7 is known to be expressed on immune cells (Malaer and Mathew, 2017). Thus, we further investigated the inflammatory responses within cells. First, we found that Slamf7 was diffusely distributed in cardiomyocytes in C57BL/6J hearts. The expression of Slamf7 increased significantly when the myocardium was subjected to IR, but this activation was suppressed by Slit2 overexpression (Figures 6A,C). These results were consistent with the Western blot and RT-qPCR findings (Figures 5A,F,G). Moreover, we found that Slit2 overexpression inhibited the IR-induced nuclear translocation of NFκB p65 in cells (mainly fibroblasts) (Figures 6B,D).

The levels of the cytokines IL-1β and IL-18 did not differ between C57BL/6J and Slit2-Tg heart perfusates at baseline. At the beginning of reperfusion, the release of IL-1β sharply increased before exhibiting a U-shaped change in C57BL/6J hearts, but Slit2-Tg hearts showed smaller increases, and the levels rapidly returned to baseline levels during reperfusion (Figure 6E). Elevations in IL-18 release were seen in C57BL/6J hearts but not in Slit2-Tg hearts at the beginning of reperfusion (Figure 6F). The above results establish that Slit2 plays an anti-inflammatory role during IR.

### Slit2 Regulates Myofilament-Associated PKC in Post-IR Hearts

**FIGURE 7:**
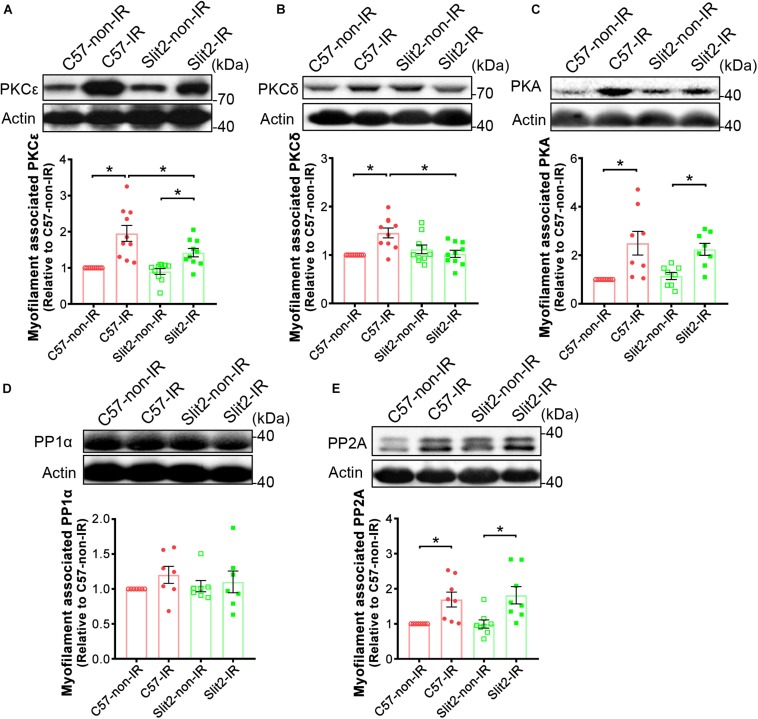
Expression of myofilament-associated PKCs, PKA, and PPs. Cardiac myofilaments were probed for **(A)** PKCε, **(B)** PKCδ, **(C)** PKA, **(D)** PP1α, and **(E)** PP2A in C57BL/6J and Slit2-Tg hearts subjected or not subjected to IR. The protein expression levels were normalized to those in non-ischemic C57BL/6J hearts. *n* = 7–10 mice per group. All data are presented as the mean ± SEM; *P < 0.05 between the groups (two-way ANOVA, Tukey’s multiple comparisons test).

In previous experiments, we found that Slit2 improved contractile function, protected cardiac tissues (Figures 1, 2), and shifted gene expression profiles to enhance the regulation of cardiac contraction (Figure 3C). Consequently, we investigated myofilament-associated PKA and PKC and their counterparts PP type 1 (PP1) and PP2A, which are known to be activated during IR and to regulate cardiac contraction (Lochner et al., 1999; Armstrong, 2004; Nicolaou et al., 2009). Here, we found that myofilament-associated PKCδ was significantly upregulated in C57BL/6J hearts after IR, but the increases in PKCδ were blocked by Slit2 post IR (Figure 7B). Slit2 did not fully block the IR-induced upregulation of PKCε in C57BL/6J mice (Figure 7A). The IR-induced increases in myofilament-associated PKA and PP2A were not altered by Slit2 overexpression post IR (Figures 7C,E). The expression of myofilament-associated PP1α was not different between any groups (Figure 7D). The above results establish that Slit2 targets myofilament-associated PKC phosphorylation signaling in the post-IR myocardium.

### Slit2 Suppresses cTnI Ser43 Phosphorylation and Maintains Myofilament Contractile Function

**FIGURE 8:**
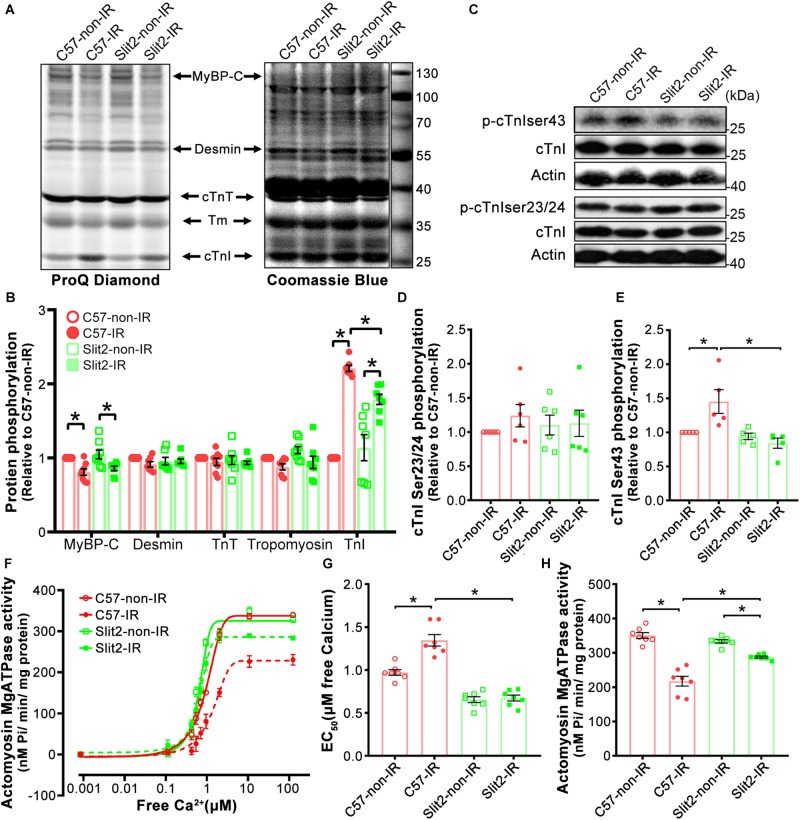
Myofilament phosphorylation and contractile properties. **(A)** ProQ phosphorylation analysis and Coomassie staining of cardiac myofilament proteins after IR. **(B)** Phosphorylation levels of MyBP-C, desmin, cTnT, and cTnI. *n* = 7–8 mice per group. **(C–E)** Representative Western blots of cTnI phosphorylation **(C)** and quantification of cTnI phosphorylation at Ser23/24 **(D)** and Ser43 **(E)**. *n* = 5–6 mice per group. **(F)** Illustrations of actomyosin MgATPase activity in each group. **(G)** Myofilament EC_50_ values (indicators of myofilament calcium sensitivity). **(H)** Maximum actomyosin MgATPase activity. *n* = 7 mice per group. All data are presented as the mean ± SEM; *P < 0.05 between the groups (two-way ANOVA, Tukey’s multiple comparisons test).

Several cardiac myofilament proteins, including cTnI, cTnT, MyBP-C, and desmin, can be targeted by protein kinases (Mohamed et al., 1998; Solaro, 2008; Streng et al., 2013). Following identification of myofilament-associated PKC regulation by Slit2, we explored myofilament phosphorylation levels and specific sites affected by Slit2 in the post-IR myocardium. We found that cTnI phosphorylation was increased in both post-IR C57BL/6J and Slit2-Tg hearts, but the increase was relatively suppressed in post-IR Slit2-Tg hearts (Figures 8A,B). MyBP-C phosphorylation was decreased in C57BL/6J and Slit2-Tg hearts after IR, and the decreases did not differ between the groups (Figures 8A,B). Desmin and cTnT did not show any differences between the groups (Figures 8A,B). Moreover, we found that the phosphorylation levels of cTnI at Ser43 were higher than baseline levels in post-IR C57BL/6J hearts, but Slit2 significantly decreased the IR-induced phosphorylation at this site (Figures 8C,E). These changes were consistent with the findings for cTnI phosphorylation. cTnI phosphorylation at Ser23/24 did not differ between any groups (Figures 8C,D). Thus, we identified that cTnI Ser43 was targeted by Slit2 during IR, and this site is known for phosphorylation by PKCs. Given the findings regarding the activation of myofilament-associated PKC, we conclude that Slit2 regulates myofilament activation at least in part by targeting PKC-dependent cTnI phosphorylation.

Myofilament contractile properties (maximum actomyosin MgATPase activity and calcium sensitivity) were examined to further explain the post-IR protective effects of Slit2. We found that while maximum actomyosin MgATPase activity did not differ among non-ischemic hearts (Figures 8F,H), Slit2-Tg hearts had higher calcium sensitivity than C57BL/6J hearts (Figure 8G). Maximum actomyosin MgATPase activity decreased significantly in both C57BL/6J and Slit2-Tg hearts post IR, but Slit2-Tg hearts maintained relatively higher contractility than C57BL/6J hearts (Figures 8F,H). The calcium sensitivity of C57BL/6J myofilaments was significantly decreased after IR, but it was not altered in post-IR Slit2 myofilaments (Figure 8G). In summary, Slit2 protects myofilament contractile function in post-IR hearts, and this effect is associated with Slit2-mediated depression of cTnI phosphorylation.

## Discussion

**FIGURE 9:**
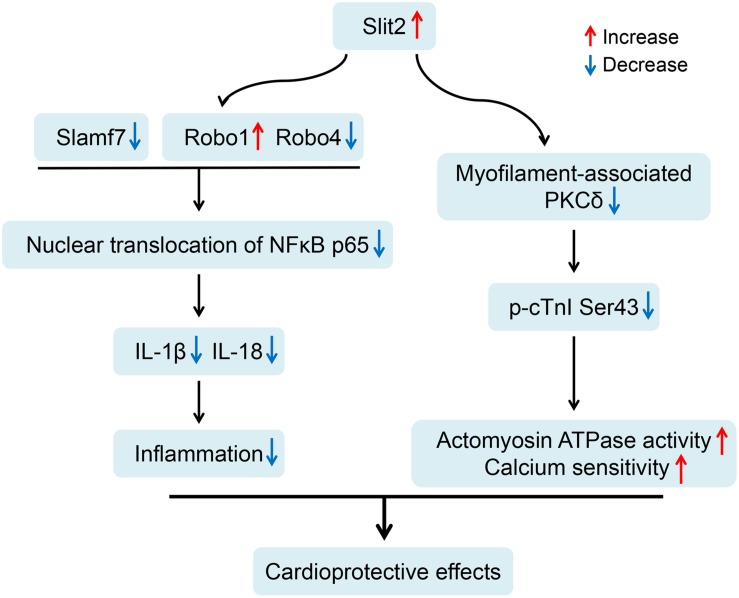
Proposed cardioprotective mechanisms of Slit2 in the post-ischemia–reperfusion (IR) myocardium. Slit2 overexpression inhibits the expression of the membrane receptors Robo4 and Slamf7 but activates Robo1, which in turn blocks the nuclear translocation of NFκB p65 and impedes the release of IL-1β and IL-18 from the cells in post-IR hearts. Next, Slit2 regulates intracellular signaling pathways to attenuate the increases in myofilament-associated PKC levels and cTnI Ser43 phosphorylation in the post-IR myocardium, maintaining myofilament contractility and calcium sensitivity.

Our current study demonstrates that Slit2 overexpression protects hearts against IR injury by maintaining postischemic contractile function and reducing tissue damage. The beneficial effects of Slit2 post IR were confirmed by evaluating cardiac contractile function, cellular structure, anti-inflammatory responses of the myocardium, and myofilament phosphorylation status and contractile properties. The proposed mechanisms for the cardioprotective effects of Slit2 in the post-IR myocardium are shown in Figure 9. Briefly, Slit2 overexpression inhibits the expression of the membrane receptors Robo4 and Slamf7 but activates Robo1, which in turn blocks the nuclear translocation of NFκB p65 and impedes the release of IL-1β and IL-18 from cells in post-IR hearts. Next, Slit2 regulates intracellular signaling pathways to attenuate the increases in myofilament-associated PKC levels and cTnI Ser43 phosphorylation in the post-IR myocardium, maintaining myofilament contractility and calcium sensitivity. Our study establishes, for the first time, that Slit2 protects hearts against IR injury and regulates myofilament phosphorylation post IR.

The Slit2-Robo signaling pathway has also been implicated in the response to IR injury in other organ systems. For example, Slit2 levels in the brain increase following ischemic injury (Park et al., 2016), and Slit2 is a crucial element in glial scar formation and brain remodeling in response to ischemic stroke (Jin et al., 2016). Slit2 has been shown to antagonize the actions of neutrophils in the kidney and protect against IR (Chaturvedi et al., 2013). The mechanisms of renal IR injury are similar to those of cardiac IR injury: ATP depletion, ROS generation, ionic dysregulation, and increased inflammatory cytokine and chemokine production. Thus, Slit2 might offer similar protection against IR injury in the heart through similar mechanisms. Moreira et al. (2014) found that changes in Slit2 levels associated with peptide treatment were correlated with cardiac recovery following sepsis, but the authors did not test whether increases in Slit2 were sufficient to mediate cardioprotection. Our current study is the first to show that increased expression of Slit2 protects against cardiac IR injury and supports the therapeutic potential of Slit2 treatment for a variety of ischemic injuries. One potential limitation for the translation of Slit2 treatment might be the inhibitory effects of Slit2 on the immune system. Interestingly, the effects of Slit2 on neutrophils in the kidney in response to IR do not negatively impact innate immune neutrophil functions, showing that Slit2-focused treatments do not increase the risk of opportunistic infections (Chaturvedi et al., 2013). Furthermore, acute application of Slit2 agonists around the time of reperfusion may be a therapeutic approach that avoids any immune complications that could limit the potential of chronic Slit2 treatment.

Investigation into the inflammatory response machinery supported our above conclusions about an antagonistic role for Slit2 in postischemic immune activation. RNA sequencing analysis showed that hearts overexpressing Slit2 were sensitive to external stimuli, resisted inflammatory insults, and exhibited improved cell survival. Among the 12 genes that were upregulated during IR, 7 were inflammatory genes (Ubc, Cd274, Ccl24, Slamf7, H2Q7, Igtp, and H2Q6) (Figure 4C). In this study, we also found that Slit2 blocked the nuclear translocation of NFκB p65 in both cardiomyocytes and fibroblasts, indicating that multiple cell types might be involved in Slit2-induced cardioprotective effects. These findings are consistent with those of Pilling et al. (2014), who reported that Slit2 has direct inhibitory effects on fibrosis. Furthermore, we found that Slamf7, a receptor expressed on natural killer (NK) cells, B cells, T cells, NK T cells, dendritic cells, and monocytes, is involved in functional regulation in the post-IR myocardium. This is the first study to show that Slamf7 is present in cardiomyocytes and is linked to IR injury and Slit2-regulated cardioprotection. It has previously been shown that cleaved Slamf7 promotes the growth of multiple myeloma (Kikuchi et al., 2020) and that inhibition of SLAMF7 blocks adhesion of multiple myeloma cells to bone marrow stromal cells (Bouchon et al., 2001; Hsi et al., 2008). Slamf7 has been found to regulate PLCγ and PI_3_K in human NK cells (Tassi and Colonna, 2005) and to be upregulated in cultured neonatal cardiomyocytes following acute cathepsin G exposure (Shukla et al., 2018). These findings suggest that Slamf7 can be present in cardiomyocytes and that it responds to various stresses. The mechanisms associated with the interaction of Slamf7 with intracellular signaling pathways and the cardiac expression/translocation of Slamf7 need to be further explored.

Roman et al. (2004) found that inhibition of PKC-mediated phosphorylation of cTnI improves cardiac performance. We observed that the levels of myofilament-associated PKCs were significantly increased by IR, but such increases were suppressed by Slit2 overexpression; consistent with these findings, Slit2 attenuated the upregulated phosphorylation of cTnI Ser43, a PKC phosphorylation site in the post-IR myocardium. Thus, our results suggest that the suppression of Slit2-regulated PKCs plays crucial roles in cardioprotection post IR. Additionally, PKC can directly interact with cytokines, as its activation is essential for the synthesis of cytokines such as TNF-α, IL-1β, and IL-6 in human monocytes (Kontny et al., 1999), suggesting that other mechanisms beyond those investigated in our study might be involved in Slit2-regulated PKC signaling. Furthermore, the levels of myofilament-associated PKA and PP2A were increased in the post-IR myocardium in C57BL/6J mice, but these changes were not modified by Slit2. This finding suggests that PKA and PP2A might not be crucial effectors of Slit2 in the postischemic heart.

Myofilament phosphorylation status is critical for normal contractile function (Verduyn et al., 2007; Kooij et al., 2010). In the present study, we found that the increases in cTnI phosphorylation post IR could be attenuated by Slit2, which supports claims that cTnI phosphorylation status is the key player in determining cardiac contractile function (McDonough and Van Eyk, 2004; Heijman et al., 2013). Furthermore, we identified the Ser43 site of cTnI as a downstream target of Slit2 and determined cTnI phosphorylation levels in post-IR Slit2-Tg hearts. Phosphorylation of cTnI Ser43/45 sites increases force in response to phenylephrine (Montgomery et al., 2002) and reduces ATPase activity (Jideama et al., 1996). Here, we found that altered cTnI phosphorylation was associated with preserved actomyosin MgATPase activity and calcium sensitivity in post-IR Slit2-Tg hearts. Thus, we clearly show that Slit2 targets myofilament phosphorylation, which plays crucial roles in maintaining myofilament contractile properties after IR injury.

In conclusion, we demonstrate that Slit2 protects cardiac function and reduces IR injury and that these effects are regulated by Slit2 interaction with membrane receptors that inhibit inflammatory responses and maintain myofilament contractile properties. This is the first study to show that Slit2 can protect hearts against IR injury and to identify that myofilament phosphorylation is regulated by Slit2.

## Limitations of the Study

The Slit2-Tg mice were constructed using a CMV promoter. Niwa et al. (1991) developed the CMV vector system, which has been widely used to stably express foreign genes in mammalian models and normal cells. However, as a non-tissue-specific promoter, CMV might drive Slit2 expression in multiple organs and multiple cell types within an organ. In this study, Slit2 overexpression was driven by the CMV promoter, suggesting that Slit2 might play roles in all types of cells residing in the heart, such as cardiomyocytes, fibroblasts, and endothelial cells. When interpreting the results of this study, we should consider the overall effects of Slit2 on cardiac tissues, not limiting our conclusions to cardiomyocytes. In addition, an isolated perfused heart model that represents an acute ischemic condition was used in this study to understand the cardioprotective mechanisms of Slit2. The effects of Slit2 during chronic ischemia need to be further investigated. It is anticipated that an *in vivo* model will help to reveal the long-term effects of Slit2.

In this study, although the contractile phenotype is consistent with phosphorylation of Ser43/45 on cardiac troponin I (cTnI), some regulation mechanisms regarding Slit2-induced myofilament phosphorylation were not clear. For example, cTnI Thr144 is phosphorylated by PKC under acidosis, a condition induced by ischemia (Engel et al., 2009). Although it exhibits functions different from those of cTnI Ser43/45 after phosphorylation, this site might contribute to the functional adaptation regulated by Slit2 post IR. Besides cTnI, other myofilaments including MyBP-C (Mohamed et al., 1998) and cTnT (Jideama et al., 1996) were targeted by PKC. Although we did not detect the overall changes in the phosphorylation levels of these myofilaments in the post-IR myocardium, PKC might collaborate with other protein kinases and phosphatases to exert the effects. Another unclear mechanism is the roles of MyBP-C dephosphorylation in the IR context. We found that MyBP-C was dephosphorylated in post-IR Slit2-Tg hearts, which contrasts with previous reports showing that MyBP-C dephosphorylation is associated with heart dysfunction (Sadayappan et al., 2006). Thus, further study is needed for evaluating the roles of the site-specific and/or PKC-dependent myofilament phosphorylation in the Slit2-regulated post-IR cardiac function.

## Data Availability Statement

The RNA sequencing data has been deposited in the Gene Expression Omnibus (accession: GSE133796).

## Ethics Statement

The animal study was reviewed and approved by the Institutional Animal Care and Use Committee (No. IACUC2017002).

## Author Contributions

FY, RH, PB, and XL designed and initiated the project. XL, SZ, WT, HC, XHL, JW, TL, and XR were responsible for the laboratory experiments, data analysis, and animal care. WP and LW provided critical comments during manuscript preparation. All authors read and approved the final manuscript.

## Conflict of Interest

The authors declare that the research was conducted in the absence of any commercial or financial relationships that could be construed as a potential conflict of interest.

## Footnotes

1 https://wwwn.cbi.nlm.nih.gov/geo/

2 https://omictools.com/tbtools-tool
