# Supplementary material for: Slit2 Protects Hearts Against Ischemia-Reperfusion Injury by Inhibiting Inflammatory Responses and Maintaining Myofilament Contractile Properties
Source: Front Physiol. 2020 Mar 27;11:228. doi: 10.3389/fphys.2020.00228 (PMC7135862; doi:10.3389/fphys.2020.00228)
Supplement: Supplementary file 1 [file Data_Sheet_1.docx]

**
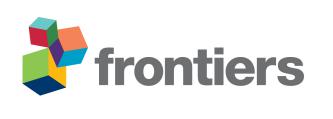
**

Supplementary Material

**Supplementary Materials and Methods**

**Semi-quantitative PCR assays**

Total RNA extracted from the left ventricle was subjected to reverse transcription and semi-quantitative PCR. The cycles were as follows: 95°C for 5 min (1 cycle); 95°C for 45 sec, 60°C for 35 sec, and 72°C for 1 min (30 cycles); and 72°C for 10 min. The PCR products were resolved on 2% agarose gels. The product bands were detected using a Gel Doc™ XR+ Gel Documentation System (Bio-Rad Laboratories Ltd., USA). Gene expression levels were normalized to the internal control gene GAPDH. All procedures were performed according to the manufacturer’s instructions, and the primer sequences were as follows: a) Slit2, forward 5’-GCGAAGCTATACAGGCTTGAT-3’ and reverse 5’-TGCAGTCGAAAAGTCCTAAGTTT-3’; b) GAPDH, forward 5’- TGGATTTGGACGCATTGGTC-3’ and reverse 5’-TTTGCACTGGTACGTGTTGAT-3’.

**Echocardiography**

C57BL/6J and Slit2-Tg mice were continuously anesthetized by inhaling 2% isoflurane and were maintained at 37°C using a heating plate. The B-mode and M-mode images were acquired with a Vevo 2100 system equipped with a 30-MHz high frequency transducer (FUJIFILM VisualSonics, Canada). HR, cardiac output (CO), left ventricular ejection fraction (LVEF), LV shortening fraction (LVFS), and LV volume at the end of diastole (LVVd) or at the end of systole (LVVs) were analyzed with Vevo 2100 Lab software (FUJIFILM, VisualSonics, Canada).

**
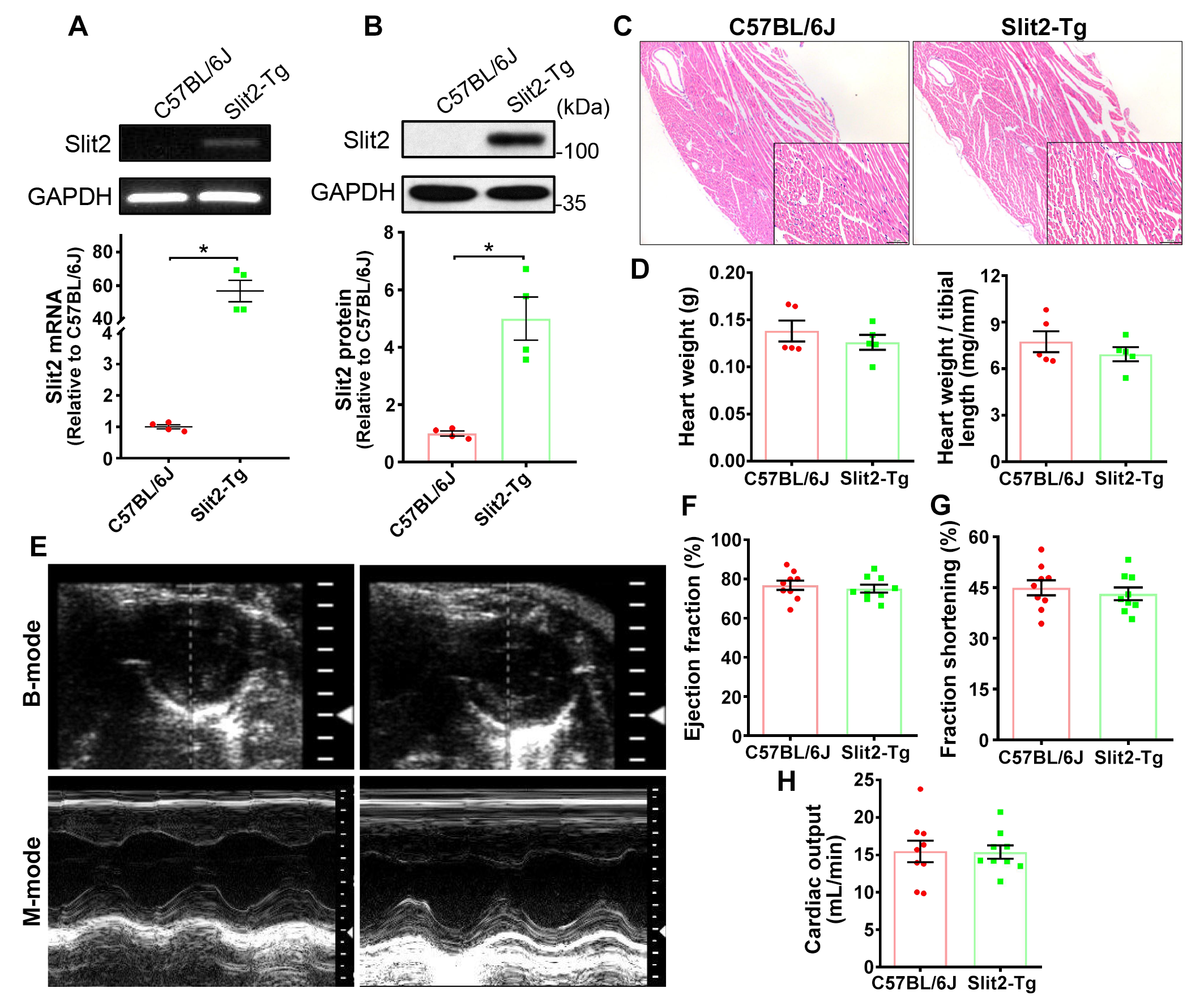
**

**Supplementary figure 1. Gene and protein expression of Slit2 in the myocardium and cardiac phenotypes of Slit2-Tg hearts.** (**A**) Semi-qPCR for Slit2 mRNA levels in C57BL/6J and Slit2-Tg hearts. A bar graph of the expression levels is shown. n = 4 mice per group. (**B**) Western blotting for Slit2 protein levels in C57BL/6J and Slit2-Tg hearts. A bar graph of the expression levels is shown. n = 4 mice per group. (**C**) Light microscopy of HE-stained heart sections from C57BL/6J and Slit2-Tg mice. (**D**) Heart weights and the ratio of the heart weight to the tibia length for C57BL/6J and Slit2-Tg mice. n = 5 mice per group. (**E**) Representative B-mode images (upper), and M-mode images (lower) of C57BL/6J and Slit2-Tg hearts. (**F-H**) Cardiac function of ejection fraction (EF%), fraction shortening (FS%) and cardiac output (CO) of Slit2 mice. n = 9 mice per group. The data are presented as the means ± SEM, *P < 0.05 vs. C57BL/6J (unpaired Student’s t-test).

**
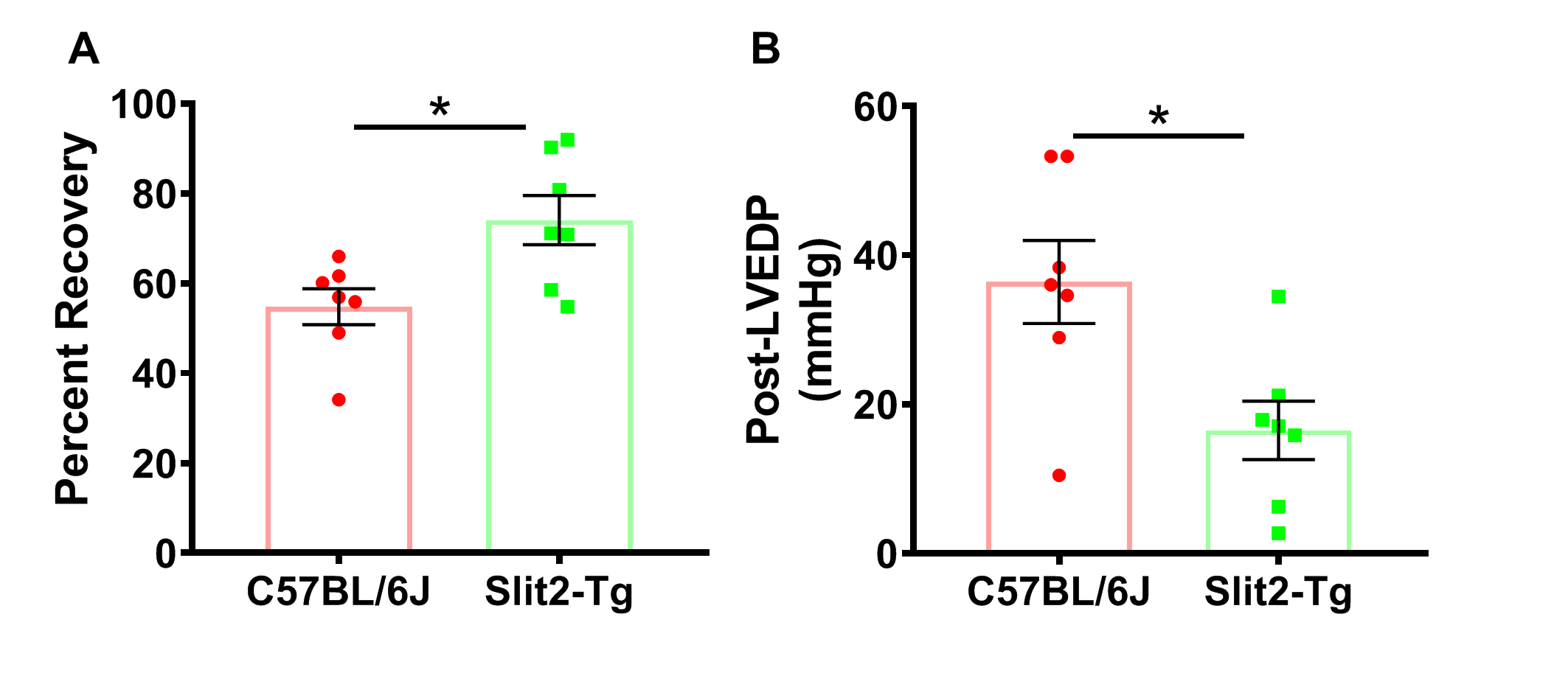
**

**Supplementary figure 2. Effects of Slit2 on post-IR cardiac function.** (**A**) Percent recovery of LVDP in post-IR C57BL/6J and Slit2-Tg hearts, (**B**) LVEDP at the end of reperfusion in C57BL/6J and Slit2-Tg hearts. n = 7 mice per group. All data are presented as the means ± SEM. *P < 0.05 vs. C57BL/6J (unpaired Student’s t-test).


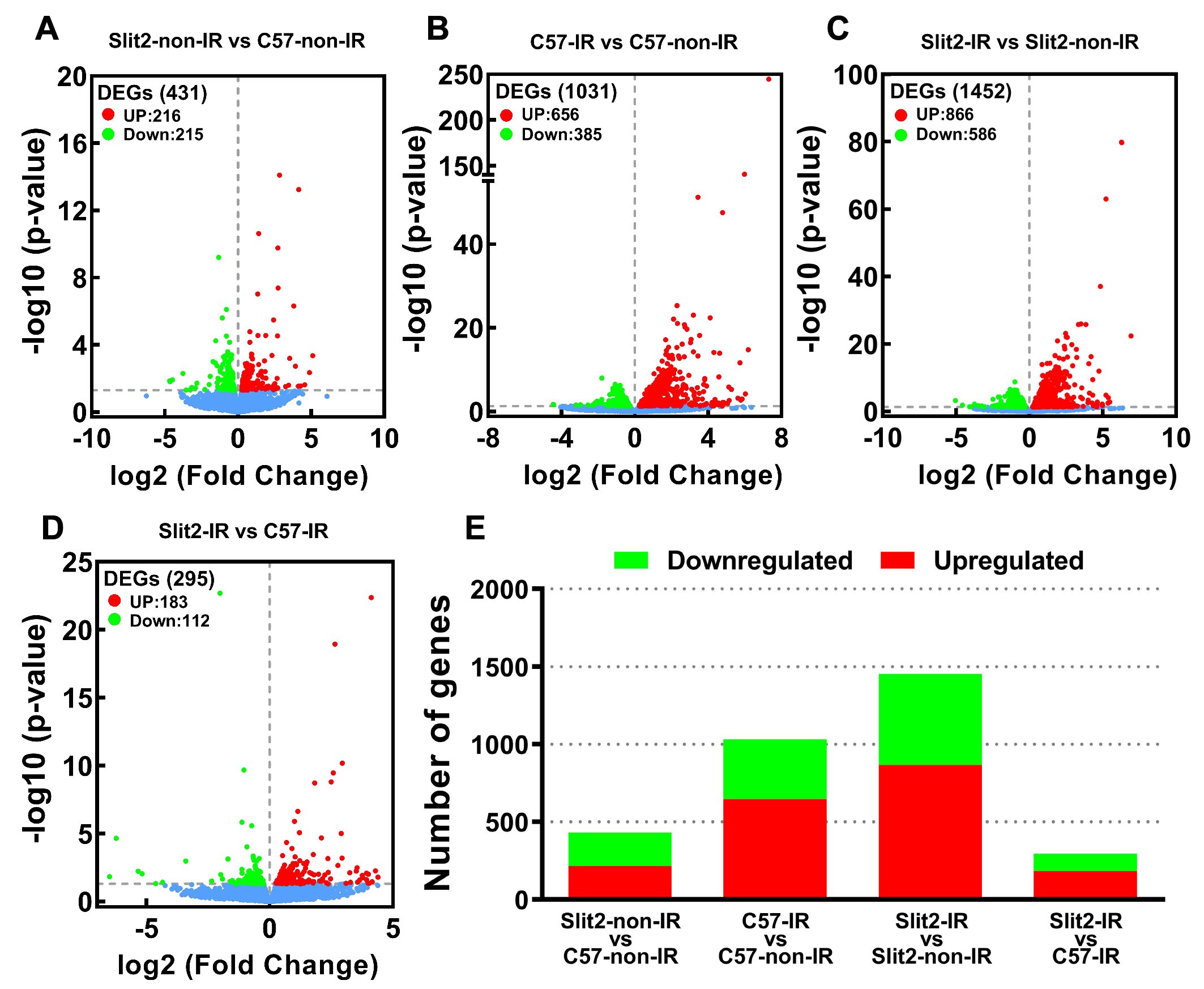


**Supplementary figure 3. Differentially expressed genes (DEGs) of Slit2-non-IR vs. C57-non-IR, C57-IR vs. C57-non-IR, Slit2-IR vs. Slit2-non-IR, and Slit2-IR vs. C57-IR.** The overall distribution of DEGs (fold change > 0 and p-Value < 0.05) is reflected by the Volcano plot. The X-axis represents the changes in gene expression in different samples, and the Y-axis represents the statistical significance of the difference in gene expression. The upregulated genes are shown in red, and the downregulated genes are shown in green. The distribution of DEGs between Slit2-non-IR vs. C57-non-IR samples (A), C57-IR vs. C57-non-IR samples (B), Slit2-IR vs. Slit2-non-IR samples (C), and Slit2-IR vs. C57-IR samples (D). (E) Total number of up- and downregulated genes in each condition.

**Supplementary Table 1: Primer sequences used for RT-qPCR and Semi-qPCR.**

| **Gene** | **Forward** | **Reverse** |
| --- | --- | --- |
| ***Primers for RT-qPCR*** | | |
| **RasGRP1** | CAAGAAGCGCAAAGTGTCCC | ACTCAAGGTAGGTGAGGTGTTC |
| **Slamf7** | GCTCGTTTCTCAACGTACATCA | TGCTGTGACTGTTAGCTGACA |
| **Robo1** | AGTTTCAAGGAGCAGACAGTGA | CAGTCCCATTTCCATCATTCTT |
| **Robo2** | GGGTTACTACATCTGCCAGGCTT | AGGTGGAGGTCTATCTGTCAAAACAT |
| **Robo4** | CTCCTCGCTGTCATCCTTAGAG | GTGTCTCCTCCCCATCACTG |
| **GAPDH** | TGGATTTGGACGCATTGGTC | TTTGCACTGGTACGTGTTGAT |
| ***Primers for Semi-qPCR*** | | |
| **Slit2** | GCGAAGCTATACAGGCTTGAT | TGCAGTCGAAAAGTCCTAAGTTT |
| **GAPDH** | TGGATTTGGACGCATTGGTC | TTTGCACTGGTACGTGTTGAT |

**Supplementary Table 2: Echocardiographic analysis of Slit2-Tg and C57BL/6J mice in vivo.**

|  | **HR (beat/min)** | **CO (mL/min)** | **LVEF%** | **LVFS%** | **LVVd (µl)** | **LVVs (µl)** |
| --- | --- | --- | --- | --- | --- | --- |
| **C57BL/6J**  **(n = 9)** | 474.3 ± 10.78 | 15.47 ± 1.437 | 76.82 ± 2.362 | 44.94 ± 2.231 | 47.20 ± 3.626 | 10.99 ± 1.483 |
| **Slit2-Tg**  **(n = 9)** | 457.1 ± 14.54 | 15.36 ± 0.902 | 75.12 ± 2.019 | 43.14 ± 1.885 | 43.82 ± 2.083 | 10.93 ± 1.043 |

All data are presented as the means ± SEM. Key: HR, heart rate; CO, cardiac output; LVEF%, left ventricle ejection fraction; LVFS%, left ventricular shortening fraction; LVVd, LV volume at the end of diastole; LVVs, LV volume at the end of systole.

**Supplementary Table 3: List of DEGs in functional enrichment analysis in Fig. 3.**

| ***Group*** | ***Term*** | ***Description*** | ***LogP*** | ***Gene***  ***Ratio*** | ***DEGs*** |
| --- | --- | --- | --- | --- | --- |
| **Slit2-non-IR vs. C57-non-IR Up** | ***Regulation of cell adhesion*** | | | | |
|  | GO:0030155 | regulation of cell adhesion | -5.836 | 22/687 | *Adipoq, Adam19, Apod, Atm, Bcl6, Cd28, Abat, Lama2, Smad3, Myoc, Pde3b, Plxna1, Sema4d, Il6st, Prnp, Ret, Abi3bp, Magi2, Rreb1, Vwc2, Dmd, Sox9* |
|  | GO:0045785 | positive regulation of cell adhesion | -3.048 | 12/412 | *Adam19, Atm, Bcl6, Cd28, Dmd, Il6st, Smad3, Myoc, Ret, Rreb1, Vwc2, Abi3bp* |
|  | GO:0010810 | regulation of cell-substrate adhesion | -2.897 | 8/211 | *Apod, Bcl6, Dmd, Smad3, Myoc, Rreb1, Abi3bp, Vwc2* |
|  | GO:0010811 | positive regulation of cell-substrate adhesion | -2.767 | 6/127 | *Dmd, Smad3, Myoc, Rreb1, Vwc2, Abi3bp* |
|  | GO:0031589 | cell-substrate adhesion | -2.665 | 10/339 | *Apod, Bcl6, Dmd, Smad3, Myoc, Cntn2, Rreb1, Fat2, Vwc2, Abi3bp* |
|  | GO:0001952 | regulation of cell-matrix adhesion | -2.264 | 5/113 | *Apod, Bcl6, Dmd, Smad3, Myoc* |
|  | GO:0001953 | negative regulation of cell-matrix adhesion | -2.199 | 3/38 | *Apod, Bcl6, Myoc* |
|  | GO:1901888 | regulation of cell junction assembly | -2.024 | 4/83 | *Apod, Gja1, Smad3, Myoc* |
|  | ***Protein kinase B signaling*** | | | | |
|  | GO:0043491 | protein kinase B signaling | -4.753 | 10/186 | *Cd28, Fgf2, Gata4, Igfbp5, Irs1, Myoc, Ret, Sox9, Magi2, Ep300* |
|  | GO:0051896 | regulation of protein kinase B signaling | -3.031 | 7/155 | *Cd28, Fgf2, Gata4, Igfbp5, Myoc, Ret, Magi2* |
|  | GO:0043501 | skeletal muscle adaptation | -2.830 | 3/23 | *Igfbp5, Myoc, Tead1* |
|  | GO:0051897 | positive regulation of protein kinase B signaling | -2.452 | 5/102 | *Cd28, Fgf2, Igfbp5, Myoc, Ret* |
|  | GO:0014068 | positive regulation of PI3K signaling | -2.261 | 4/71 | *Cd28, Myoc, Sema4d, Sox9* |
|  | GO:0014065 | PI3K signaling | -2.054 | 5/127 | *Cd28, Irs1, Myoc, Sema4d, Sox9* |
|  | ***Response to hypoxia*** | | | | |
|  | GO:0001666 | response to hypoxia | -4.409 | 10/204 | *Aldh3a1, Atm, Gata6, Smad3, Ryr2, Raf1, Pdk1, Cpeb2, Abat, Ep300* |
|  | GO:0036293 | response to decreased oxygen levels | -3.974 | 10/230 | *Aldh3a1, Atm, Gata6, Smad3, Ryr2, Raf1, Pdk1, Cpeb2, Abat, Ep300* |
|  | mmu04068 | FoxO signaling pathway | -3.447 | 7/132 | *Atm, Bcl6, Irs1, Smad3, Pck1, Raf1, Ep300* |
|  | GO:0070482 | response to oxygen levels | -3.265 | 10/282 | *Aldh3a1, Atm, Gata6, Smad3, Ryr2, Raf1, Pdk1, Cpeb2, Abat, Ep300* |
|  | mmu04371 | Apelin signaling pathway | -2.554 | 6/140 | *Smad3, Mef2c, Pde3b, Ryr2, Plin1, Raf1* |
|  | mmu04024 | cAMP signaling pathway | -2.440 | 7/197 | *Acox1, Camk2a, Pde3b, Ryr2, Sox9, Ep300, Raf1* |
|  | ***Organic acid catabolic process*** | | | | |
|  | GO:0016054 | organic acid catabolic process | -4.234 | 10/214 | *Acox1, Adipoq, Fgf2, Irs1, Pah, Pck1, Abcd2, Echdc1, Aldh1l2, Abat* |
|  | GO:0046395 | carboxylic acid catabolic process | -4.234 | 10/214 | *Acox1, Adipoq, Fgf2, Irs1, Pah, Pck1, Abcd2, Echdc1, Aldh1l2, Abat* |
|  | GO:0072329 | monocarboxylic acid catabolic process | -3.887 | 7/112 | *Acox1, Adipoq, Irs1, Pck1, Abcd2, Echdc1, Abat* |
|  | GO:0006006 | glucose metabolic process | -3.823 | 9/195 | *Adipoq, Apod, Irs1, Ncoa2, Pcx, Pdk1, Ppp1r3b, Pck1, Ep300* |
|  | GO:0044242 | cellular lipid catabolic process | -3.756 | 9/199 | *Acox1, Adipoq, Sorl1, Abcd2, Echdc1, Gpcpd1, Irs1, Pck1, Pnpla3* |
|  | GO:0016042 | lipid catabolic process | -3.590 | 11/306 | *Acox1, Adipoq, Irs1, Pck1, Pde3b, Sorl1, Abcd2, Echdc1, Gpcpd1, Plin1, Pnpla3* |
|  | GO:0009062 | fatty acid catabolic process | -3.478 | 6/93 | *Acox1, Adipoq, Irs1, Pck1, Abcd2, Echdc1* |
|  | GO:0019318 | hexose metabolic process | -3.377 | 9/224 | *Adipoq, Apod, Irs1, Ncoa2, Pck1, Pcx, Pdk1, Ppp1r3b, Ep300* |
|  | GO:0034440 | lipid oxidation | -3.308 | 6/100 | *Acox1, Adipoq, Apod, Irs1, Abcd2, Echdc1* |
|  | GO:0006635 | fatty acid beta-oxidation | -3.267 | 5/67 | *Acox1, Adipoq, Irs1, Abcd2, Echdc1* |
| **Slit2-non-IR vs. C57-non-IR Down** | ***Positive regulation of glycogen metabolic process*** | | | | |
|  | GO:0070875 | positive regulation of glycogen metabolic process | -4.477 | 4/19 | *Hmgb1, C1qtnf2, Gck, Ppp1r3e* |
|  | GO:0032881 | regulation of polysaccharide metabolic process | -4.125 | 5/44 | *Hmgb1, Pdgfb, C1qtnf2, Gck, Ppp1r3e* |
|  | GO:0062013 | positive regulation of small molecule metabolic process | -3.771 | 8/155 | *Bcl2l1, Hmgb1, Pdgfb, Plin5, C1qtnf2, Gper1, Gck, Ppp1r3e* |
|  | GO:0045913 | positive regulation of carbohydrate metabolic process | -3.707 | 6/84 | *Hmgb1, Pdgfb, C1qtnf2, Gper1, Gck, Ppp1r3e* |
|  | GO:0070873 | regulation of glycogen metabolic process | -3.401 | 4/35 | *Hmgb1, C1qtnf2, Gck, Ppp1r3e* |
|  | GO:0010676 | positive regulation of cellular carbohydrate metabolic process | -3.349 | 5/64 | *Hmgb1, C1qtnf2, Gper1, Gck, Ppp1r3e* |
|  | GO:0032885 | regulation of polysaccharide biosynthetic process | -3.307 | 4/37 | *Pdgfb, C1qtnf2, Gck, Ppp1r3e* |
|  | GO:0043255 | regulation of carbohydrate biosynthetic process | -3.229 | 6/103 | *Pdgfb, C1qtnf12, C1qtnf2, Gper1, Gck, Ppp1r3e* |
|  | GO:0045725 | positive regulation of glycogen biosynthetic process | -3.220 | 3/17 | *C1qtnf2, Gck, Ppp1r3e* |
|  | GO:0010907 | positive regulation of glucose metabolic process | -3.056 | 4/43 | *Hmgb1, C1qtnf2, Gck, Ppp1r3e* |
|  | ***PPAR signaling pathway*** | | | | |
|  | mmu03320 | PPAR signaling pathway | -3.679 | 6/85 | *Fabp4, Aqp7, Angptl4, Plin5, Pck2, Scd4* |
|  | GO:0046460 | neutral lipid biosynthetic process | -3.262 | 4/38 | *Ang, Lpin3, Plin5, Pck2* |
|  | GO:0046463 | acylglycerol biosynthetic process | -3.262 | 4/38 | *Ang, Lpin3, Plin5, Pck2* |
|  | GO:0006639 | acylglycerol metabolic process | -2.845 | 6/122 | *Ang, Mgll, Dgke, Lpin3, Plin5, Pck2* |
|  | GO:0006638 | neutral lipid metabolic process | -2.809 | 6/124 | *Ang, Mgll, Dgke, Lpin3, Plin5, Pck2* |
|  | GO:0045017 | glycerolipid biosynthetic process | -2.528 | 6/141 | *Ang, Ajuba, Pdgfb, Lpin3, Plin5, Pck2* |
|  | GO:0008610 | lipid biosynthetic process | -2.455 | 13/547 | *Ang, Cyp1a1, Ajuba, Pdgfb, Lpin3, Prxl2b, Mgll, Plin5, Fam57b, Enho, Pck2, Gper1, Scd4* |
|  | GO:0006631 | fatty acid metabolic process | -2.322 | 10/377 | *Fabp4, Cyp1a1, Slc25a17, Mgll, Lpin3, Prxl2b, Plin5, C1qtnf2, Pck2, Scd4* |
|  | GO:0019432 | triglyceride biosynthetic process | -2.294 | 3/35 | *Lpin3, Plin5, Pck2* |
|  | GO:0072329 | monocarboxylic acid catabolic process | -2.271 | 5/112 | *Slc25a17, Lpin3, Plin5, Hoga1, Pck2* |
|  | ***Regulation of inflammatory response*** | | | | |
|  | GO:0050727 | regulation of inflammatory response | -3.045 | 11/354 | *Fabp4, Cd59a, S100a8, Sema7a, Gper1, Il20rb, C1qtnf12, Mgll, Il17d, Cd59b, Gpx4* |
|  | GO:0031347 | regulation of defense response | -2.288 | 14/640 | *Fabp4, Cd59a, Hmgb1, S100a8, Sema7a, Il17d, Mgll, C1qtnf12, Gper1, Apobec3, Il20rb, Cd59b, Gpx4, Ifi208* |
|  | ***Regulation of release of cytochrome c from mitochondria*** | | | | |
|  | GO:0090199 | regulation of release of cytochrome c from mitochondria | -2.909 | 4/47 | *Bcl2l1, Plaur, Fam162a, Gper1* |
|  | GO:0090200 | positive regulation of release of cytochrome c from mitochondria | -2.572 | 3/28 | *Plaur, Fam162a, Gper1* |
|  | GO:0001836 | release of cytochrome c from mitochondria | -2.516 | 4/60 | *Bcl2l1, Plaur, Fam162a, Gper1* |
| **Slit2-IR vs. C57-IR Up** | ***Collagen chain trimerization*** | | | | |
|  | R-MMU-8948216 | Collagen chain trimerization | -10.444 | 9/39 | *Col11a1, Col12a1, Col15a1, Col4a3, Col5a1, Col6a2, Col1a1, Col6a6, Col8a2* |
|  | R-MMU-2022090 | Assembly of collagen fibrils and other multimeric structures | -10.359 | 10/56 | *Col11a1, Col12a1, Col15a1, Col4a3, Col5a1, Col6a2, Col1a1, Loxl2, Col6a6, Col8a2* |
|  | R-MMU-1442490 | Collagen degradation | -9.018 | 9/55 | *Col11a1, Col12a1, Col15a1, Col4a3, Col5a1, Col6a2, Col1a1, Col6a6, Col8a2* |
|  | R-MMU-1474244 | Extracellular matrix organization | -8.777 | 16/270 | *Ddr1, Capn3, Col11a1, Col12a1, Col15a1, Col4a3, Col5a1, Col6a2, Col1a1, Hspg2, Itgb6, Lama2, Loxl2, Itga9, Col6a6, Col8a2* |
|  | R-MMU-1650814 | Collagen biosynthesis and modifying enzymes | -8.735 | 9/59 | *Col11a1, Col12a1, Col15a1, Col4a3, Col5a1, Col6a2, Col1a1, Col6a6, Col8a2* |
|  | R-MMU-1474290 | Collagen formation | -8.717 | 10/81 | *Col11a1, Col12a1, Col15a1, Col4a3, Col5a1, Col6a2, Col1a1, Loxl2, Col6a6, Col8a2* |
|  | R-MMU-216083 | Integrin cell surface interactions | -8.001 | 9/71 | *Col4a3, Col5a1, Col6a2, Col1a1, Hspg2, Itgb6, Itga9, Col6a6, Col8a2* |
|  | R-MMU-1474228 | Degradation of the extracellular matrix | -7.808 | 11/129 | *Col11a1, Col12a1, Col15a1, Col4a3, Col5a1, Col6a2, Col1a1, Capn3, Hspg2, Col6a6, Col8a2* |
|  | mmu04974 | Protein digestion and absorption | -7.085 | 9/90 | *Col11a1, Col12a1, Col15a1, Col4a3, Atp1a4, Col5a1, Col6a2, Col1a1, Col6a6* |
|  | R-MMU-3000178 | ECM proteoglycans | -7.040 | 7/44 | *Col5a1, Col6a2, Col1a1, Hspg2, Itgb6, Itga9, Col6a6* |
|  | ***Regulation of ion transport*** | | | | |
|  | GO:0043269 | regulation of ion transport | -5.839 | 21/733 | *Cacna1g, Camk2a, Capn3, Casq1, Dpp6, Drd2, Gnas, Kcna1, Kcna7, Pla2g6, Kcne1, Shank2, Nos1, Hcn2, Nrxn1, Prnp, Stc2,Kcnq4, Rab3b, Gsg1l, Scn4b* |
|  | GO:0034765 | regulation of ion transmembrane transport | -5.393 | 16/478 | *Capn3, Casq1, Kcna1, Kcna7, Kcne1, Shank2, Dpp6, Drd2, Hcn2, Nos1, Nrxn1, Prnp, Pla2g6, Kcnq4, Gsg1l, Scn4b* |
|  | GO:0098655 | cation transmembrane transport  regulation of transmembrane transport | -5.070 | 19/691 | *Cacna1g, Capn3, Casq1, Kcna1, Kcna7, Dpp6, Drd2, Hcn2, Kcne1, Nos1, Nrxn1, Prnp, Ryr1, Atp1a4, Pla2g6, Kcnq4, Shank2, Gsg1l, Scn4b* |
|  | GO:0034762 | regulation of cation | -5.056 | 17/568 | *Capn3, Casq1, Dpp6, Drd2, Hcn2, Irs1, Kcna1, Kcna7, Kcne1, Nos1, Nrxn1, Kcnq4, Shank2, Prnp, Pla2g6, Gsg1l, Scn4b* |
|  | GO:1904062 | transmembrane transport | -4.962 | 13/349 | *Capn3, Casq1, Kcna1, Kcne1, Pla2g6, Shank2, Dpp6, Drd2, Nos1, Nrxn1, Prnp, Gsg1l, Scn4b* |
|  | GO:0098660 | inorganic ion transmembrane transport | -4.806 | 18/657 | *Cacna1g, Capn3, Casq1, Dpp6, Drd2, Gabra1, Gabrb2, Kcna1, Kcna7, Kcne1, Atp1a4, Kcnq4, Hcn2, Nos1, Prnp, Ryr1, Pla2g6, Scn4b* |
|  | GO:0010959 | regulation of metal ion transport | -4.738 | 14/422 | *Cacna1g, Camk2a, Capn3, Casq1, Dpp6, Drd2, Gnas, Kcna1, Kcne1, Nos1, Prnp, Stc2, Pla2g6, Scn4b* |
|  | GO:0043270 | positive regulation of ion transport | -4.622 | 12/322 | *Camk2a, Capn3, Casq1, Kcna1, Kcne1, Pla2g6, Dpp6, Drd2, Gnas, Nos1, Rab3b, Scn4b* |
|  | GO:0032412 | regulation of ion transmembrane transporter activity | -4.135 | 10/254 | *Casq1, Kcna1, Kcne1, Pla2g6, Shank2, Gsg1l, Drd2, Nrxn1, Prnp, Scn4b* |
|  | GO:0098662 | inorganic cation transmembrane transport | -4.069 | 16/614 | *Cacna1g, Capn3, Casq1, Kcna1, Kcna7, Kcne1, Dpp6, Drd2, Hcn2, Nos1, Atp1a4, Pla2g6, Prnp, Kcnq4, Ryr1, Scn4b* |
|  | ***Regulation of system process*** | | | | |
|  | GO:0044057 | regulation of system process | -5.361 | 18/598 | *Cacna1g, Casq1, Fgf10, Kcna1, Kcne1, Nrxn1, Gnas, Cux2, Drd2, Dvl1, Edn2, Nos1, Shank2, Ryr1, Ccn4, Irx5, Myh7, Scn4b* |
|  | GO:0003012 | muscle system process | -5.096 | 14/392 | *Cacna1g, Casq1, Kcna1, Kcne1, Pla2g6, Drd2, Edn2, Nos1, Ryr1, Ccn4, Gatm, Obsl1, Myh7, Scn4b* |
|  | GO:0006936 | muscle contraction | -5.089 | 12/289 | *Cacna1g, Casq1, Drd2, Edn2, Kcna1, Kcne1, Nos1, Ryr1, Pla2g6, Obsl1, Myh7, Scn4b* |
|  | GO:0002027 | regulation of heart rate | -4.801 | 7/93 | *Cacna1g, Drd2, Edn2, Kcne1, Myh7, Scn4b, Irx5* |
|  | GO:0008016 | regulation of heart contraction | -3.783 | 8/179 | *Cacna1g, Drd2, Edn2, Kcne1, Nos1, Irx5, Myh7, Scn4b* |
|  | GO:0060047 | heart contraction | -3.275 | 8/213 | *Cacna1g, Drd2, Edn2, Kcne1, Nos1, Irx5, Myh7, Scn4b* |
|  | GO:0003015 | heart process | -3.157 | 8/222 | *Cacna1g, Drd2, Edn2, Kcne1, Nos1, Irx5, Myh7, Scn4b* |
|  | GO:0070252 | actin-mediated cell contraction | -3.029 | 5/87 | *Cacna1g, Kcne1, Nos1, Myh7, Scn4b* |
|  | GO:1903522 | regulation of blood circulation | -2.927 | 8/241 | *Cacna1g, Drd2, Edn2, Kcne1, Nos1, Irx5, Myh7, Scn4b* |
|  | mmu04261 | Adrenergic signaling in cardiomyocytes | -2.760 | 6/147 | *Camk2a, Gnas, Kcne1, Atp1a4, Myh7, Scn4b* |
|  | ***Response to mechanical stimulus*** | | | | |
|  | GO:0009612 | response to mechanical stimulus | -4.591 | 9/179 | *Col11a1, Col1a1, Drd2, Kcna1, Ankrd2, Ankrd1, Nos1, Nrxn1, Tmem150c* |
|  | GO:0071260 | cellular response to mechanical stimulus | -2.697 | 4/62 | *Col1a1, Nos1, Ankrd1, Tmem150c* |
|  | GO:0071496 | cellular response to external stimulus | -2.474 | 8/285 | *Cdkn2b, Col1a1, Nos1, Ankrd1, Sfrp2, Trp53, Ryr1, Tmem150c* |
|  | GO:0071214 | cellular response to abiotic stimulus | -2.411 | 8/292 | *Capn3, Col1a1, Kcne1, Sfrp2, Ankrd1, Trp53, Nos1, Tmem150c* |
|  | GO:0104004 | cellular response to environmental stimulus | -2.411 | 8/292 | *Capn3, Col1a1, Kcne1, Ankrd1, Sfrp2, Trp53, Nos1, Tmem150c* |
|  | ***Potassium ion transport*** | | | | |
|  | GO:0006813 | potassium ion transport | -4.434 | 10/234 | *Dpp6, Drd2, Hcn2, Kcna1, Kcna7, Kcne1, Nos1, Prnp, Atp1a4, Kcnq4* |
|  | GO:0071804 | cellular potassium ion transport | -3.591 | 8/191 | *Dpp6, Kcna1, Kcna7, Kcne1, Atp1a4, Kcnq4, Hcn2, Prnp* |
|  | GO:0071805 | potassium ion transmembrane transport | -3.591 | 8/191 | *Dpp6, Kcna1, Kcna7, Kcne1, Atp1a4, Kcnq4, Hcn2, Prnp* |
|  | GO:0043266 | regulation of potassium ion transport | -3.526 | 6/105 | *Dpp6, Drd2, Kcna1, Kcne1, Nos1, Prnp* |
|  | GO:0015672 | monovalent inorganic cation transport | -3.221 | 12/455 | *Dpp6, Drd2, Gnas, Hcn2, Kcna1, Kcna7, Kcne1, Nos1, Prnp, Atp1a4, Kcnq4, Scn4b* |
|  | R-MMU-1296071 | Potassium Channels | -2.878 | 5/94 | *Gnb3, Hcn2, Kcna1, Kcna7, Kcnq4* |
|  | GO:0035637 | multicellular organismal signaling | -2.852 | 6/141 | *Cacna1g,Dpp6,Kcna1,Kcne1,Nfasc,Scn4b* |
|  | GO:1904064 | positive regulation of cation transmembrane transport | -2.535 | 6/163 | *Capn3, Casq1, Dpp6, Kcna1, Kcne1, Nos1* |
|  | GO:0034764 | positive regulation of transmembrane transport | -2.383 | 7/233 | *Capn3, Casq1, Dpp6, Irs1, Kcna1, Kcne1, Nos1* |
|  | GO:0034767 | positive regulation of ion transmembrane transport | -2.336 | 6/179 | *Capn3, Casq1, Dpp6, Kcna1, Kcne1, Nos1* |
|  | ***Ion homeostasis*** | | | | |
|  | R-MMU-5578775 | Ion homeostasis | -4.118 | 5/51 | *Camk2a, Casq1, Nos1, Ryr1, Atp1a4* |
|  | R-MMU-5576891 | Cardiac conduction | -3.178 | 6/122 | *Camk2a, Casq1, Nos1, Ryr1, Atp1a4, Scn4b* |
|  | R-MMU-397014 | Muscle contraction | -2.371 | 6/176 | *Camk2a, Casq1, Nos1, Ryr1, Atp1a4, Scn4b* |
| **Slit2-IR vs. C57-IR Down** | ***Regulation of immune effector process*** | | | | |
|  | GO:0002697 | regulation of immune effector process | -6.762 | 13/422 | *Ager, Cd59a, Cd84, H2-Q7, Hmgb1, Il5, Itgam, Gbp4, Rasgrp1, Shld1, Apobec3, H2-Q6, Cd59b* |
|  | GO:0001906 | cell killing | -4.504 | 7/179 | *Ager, Cd59a, H2-Q7, Rasgrp1, Rpl30, H2-Q6, Cd59b* |
|  | GO:0002703 | regulation of leukocyte mediated immunity | -4.439 | 8/251 | *Ager,Cd84,H2-Q7,Hmgb1,Itgam,Rasgrp1,Shld1, H2-Q6* |
|  | GO:0031341 | regulation of cell killing | -4.309 | 6/131 | *Ager, Cd59a, H2-Q7, Rasgrp1, H2-Q6, Cd59b* |
|  | GO:0002699 | positive regulation of immune effector process | -3.505 | 7/259 | *Cd84, H2-Q7, Il5, Itgam, Rasgrp1, H2-Q6, Shld1* |
|  | GO:0002706 | regulation of lymphocyte mediated immunity | -3.355 | 6/196 | *Ager, H2-Q7, Hmgb1, Rasgrp1, Shld1, H2-Q6* |
|  | GO:0002819 | regulation of adaptive immune response | -3.218 | 6/208 | *Ager, H2-Q7, Hmgb1, Cd274, Shld1, H2-Q6* |
|  | GO:0050778 | positive regulation of immune response | -2.917 | 11/747 | *Cd59a, H2-Q7, Hmgb1, Itgam, Rasgrp1, Irgm2, Cd274, Shld1, H2-Q6, Gbp5, Cd59b* |
|  | GO:0002709 | regulation of T cell mediated immunity | -2.888 | 4/95 | *Ager, H2-Q7, Hmgb1, H2-Q6* |
|  | mmu04514 | Cell adhesion molecules (CAMs) | -2.807 | 5/169 | *H2-Q7, Itgam, Cd274, H2-Q6, Lrrc4c* |
|  | ***Response to interferon-gamma*** | | | | |
|  | GO:0034341 | response to interferon-gamma | -6.383 | 8/137 | *H2-Q7, Gbp4, Ccl6, Irgm2, Ccl24, Ifitm1, Gbp5, Gbp9* |
|  | GO:0071346 | cellular response to interferon -gamma | -5.766 | 7/115 | *H2-Q7, Gbp4, Ccl6, Irgm2, Ccl24, Gbp5, Gbp9* |
|  | ***Inflammatory response*** | | | | |
|  | GO:0006954 | inflammatory response | -5.093 | 14/691 | *Ager, Cd59a, Ednra, Hmgb1, Eif2ak1, Rasgrp1, Itgam, Serpina3n, Scgb1a1, Cxcl13, Ccl24, Ccl6, Gbp5, Cd59b* |
|  | GO:0031347 | regulation of defense response | -4.101 | 12/640 | *Ager, Cd59a, Ednra, Hmgb1, Rasgrp1, Irgm2, Gbp4, Scgb1a1, Ccl24, Apobec3, Gbp5, Cd59b* |
|  | GO:0030593 | neutrophil chemotaxis | -3.847 | 5/100 | *Ednra, Itgam, Ccl6, Cxcl13, Ccl24* |
|  | GO:1990266 | neutrophil migration | -3.495 | 5/119 | *Ednra, Itgam, Ccl6, Cxcl13, Ccl24* |
|  | GO:0071621 | granulocyte chemotaxis | -3.428 | 5/123 | *Ednra, Itgam, Ccl6, Cxcl13, Ccl24* |
|  | GO:0032101 | regulation of response to external stimulus | -3.355 | 12/771 | *Ager, Cd59a, Ednra, Hmgb1, cgb1a1, Cxcl13, Cd84, Gbp4, SCcl24, Apobec3, Gbp5, Cd59b* |
|  | GO:0097529 | myeloid leukocyte migration | -3.229 | 6/207 | *Ager, Ednra, Itgam, Ccl6, Cxcl13, Ccl24* |
|  | GO:0030595 | leukocyte chemotaxis | -3.175 | 6/212 | *Ednra, Itgam, Ccl6, Trpm2, Cxcl13, Ccl24* |
|  | GO:0060326 | cell chemotaxis | -3.125 | 7/300 | *Ednra, Hmgb1, Itgam, Ccl24, Trpm2, Cxcl13, Ccl6* |
|  | GO:0006935 | chemotaxis | -3.110 | 10/597 | *Ager, Ednra, Hmgb1, Hoxa1, Itgam, Ccl6, Trpm2, Cxcl13, Ccl24, Lrtm1* |
|  | ***Monocyte chemotactic protein-1 production*** | | | | |
|  | GO:0071605 | monocyte chemotactic protein-1 production | -3.894 | 3/20 | *Ager, Cd84, Hmgb1* |
|  | GO:0071637 | regulation of monocyte chemotactic protein-1 production | -3.894 | 3/20 | *Ager, Cd84, Hmgb1* |
|  | GO:0032693 | negative regulation of interleukin-10 production | -3.828 | 3/21 | *Ager, Cd84, Cd274* |
|  | R-MMU-445989 | TAK1 activates NFkB by phosphorylation and activation of IKKs complex | -3.402 | 3/29 | *Ager, Hmgb1, Ubc* |
|  | GO:0050714 | positive regulation of protein secretion | -3.108 | 7/302 | *Ager, Cd84, Hmgb1, Il5, Rasgrp1, Trpm2, Cd274* |
|  | GO:0002831 | regulation of response to biotic stimulus | -3.076 | 5/147 | *Cd84, Hmgb1, Gbp4, Cd274, Apobec3* |
|  | GO:0002275 | myeloid cell activation involved in immune response | -3.068 | 4/85 | *Cd84, Hmgb1, Itgam, Rasgrp1* |
|  | GO:0050715 | positive regulation of cytokine secretion | -3.037 | 5/150 | *Ager, Cd84, Hmgb1, Rasgrp1, Cd274* |
|  | GO:0002793 | positive regulation of peptide secretion | -2.877 | 7/331 | *Ager, Cd84, Hmgb1, Il5, Rasgrp1, Trpm2, Cd274* |
|  | R-MMU-9020702 | Interleukin-1 signaling | -2.680 | 3/51 | *Ager, Hmgb1, Ubc* |
|  | ***Regulation of leukocyte activation*** | | | | |
|  | GO:0002694 | regulation of leukocyte activation | -2.808 | 10/655 | *Ager, Itgam, Rasgrp1, Scgb1a1, Cd84, Cd274, Shld1, Il5, Slamf7, Cd59b* |
|  | GO:0050865 | regulation of cell activation | -2.615 | 10/696 | *Ager, Itgam, Rasgrp1, Scgb1a1, Cd274, Shld1, Cd84, Il5, Slamf7, Cd59b* |
|  | GO:0098609 | cell-cell adhesion | -2.492 | 10/724 | *Ager, Itgam, Rasgrp1, Scgb1a1, Cxcl13, Cd274, Crb1, Ccm2l, Lrrc4c, Cd59b* |
|  | GO:0022407 | regulation of cell-cell adhesion | -2.447 | 7/395 | *Ager, Rasgrp1, Scgb1a1, Cxcl13, Cd274, Ccm2l, Cd59b* |
|  | GO:0042098 | T cell proliferation | -2.434 | 5/206 | *Ager, Itgam, Scgb1a1, Cd274, Cd59b* |
|  | GO:0046651 | lymphocyte proliferation | -2.351 | 6/309 | *Ager, Il5, Itgam, Scgb1a1, Cd274, Cd59b* |
|  | GO:0032943 | mononuclear cell proliferation | -2.337 | 6/311 | *Ager, Il5, Itgam, Scgb1a1, Cd274, Cd59b* |
|  | GO:0050671 | positive regulation of lymphocyte proliferation | -2.323 | 4/136 | *Ager, Il5, Cd274, Cd59b* |
|  | GO:0032946 | positive regulation of mononuclear cell proliferation | -2.301 | 4/138 | *Ager, Il5, Cd274, Cd59b* |
|  | GO:0007159 | leukocyte cell-cell adhesion | -2.284 | 6/319 | *Ager, Itgam, Rasgrp1, Scgb1a1, Cd274, Cd59b* |

**Supplementary Table 4: List of differentially expressed genes (****DEGs) in Venn diagram in Fig. 4.**

| ***Group/Genes (N)*** | ***List of DEGs*** |
| --- | --- |
| **Slit2-non-IR vs. C57-non-IR All / (431)** | *Plin1, Retn, Hamp, Tmem45b, Pcna-ps2, Gucy2e, Cfd, Akap2, Cidec, Ifi202b, Slc7a11, Vwc2, 1810041L15Rik, Fer1l6, Cyp2f2, Ripk4, Ccdc65, Tmem236, Selenbp2, Pah, Ctf2, Adipoq, Bex1, Hist2h4, Eif3j2, Col11a1, Fat2, Pck1, Kcnq5, Itpka, Elfn2, Gnat3, Hp, Gabra1, Fst, Pianp, Rs1, Efhc1, Klk1b26, Lrrc75b, Zfp969, Wdfy1, Hcn1, Rab4a, Pnpla3, Itih2, Rph3al, Stab2, C1qtnf6, Aldh3a1, Paqr6, Plekhh1, Rgs1, Pcdhga1, Kif5c, Glcci1, Irs1, Abca12, Scn3b, Gatm, Helt, Tmem52, Olfml2b, Bcl6, Armcx4, Gchfr, Cntn2, Neb, Ppp1r3b, Lilr4b, Aldh1l2, Cntn5, Sox9, Myoc, Stc2, Tmem150c, Rpl22l1, Abcd2, Glt8d2, Sorl1, Ano5, Pdlim4, BC067074, Pde3b, Rpusd2, Ppargc1b, Apod, Btbd11, A930018M24Rik, Best3, Fancl, 1110002E22Rik, Kbtbd13, Abi3bp, Prg4, Snx30, Prox1, Magi3, Klhl33, Ano4, Wdr70, Mme, Cd28, Psph, Kcnv2, Tmem179, Gja1, Camk2a, Ret, Echdc1, Magi2, Sh3rf2, Zfp106, Cdon, Tmcc1, Atm, Ypel2, Lum, Gpcpd1, Asap3, Pde1c, Cecr2, Col14a1, Sptb, Zfpm2, Vps13c, Mlh3, Gclm, Cpeb2, Igfbp5, Abat, Tmem87a, Kansl1l, Cacnb2, Adam19, Atg9a, Lonrf1, Tbx5, Asb15, Fktn, Tbx20, Gpr157, Sox6, Tmtc1, Gfra1, Fgf2, Dmd, Pcx, Sema4d, Ryr2, Kcnq1, Trabd2b, Tead1, mt-Atp8, Dennd4c, Ern1, Gata4, Zfp395, Gata6, Pard3b, Arhgap32, Dvl1, Plxna1, Cep350, Ppm1k, Cxcl14, Lama2, Fbxw7, Kcnj5, Raf1, Synpo2, Corin, Steap3, Strn, Xirp1, Il6st, Cxxc5, Raph1, Cobll1, Helz, Gpc1, Smad3, Tln2, Ncoa2, Atf7ip, Mef2c, Ep300, Cacna2d1, Kdm7a, Ankrd17, Qk, Rbm20, Agl, Rreb1, Alpk2, Ascc2, Map7, Pitrm1, Otud4, Ankhd1, Acadsb, Txlnb, Map3k5, Crhr2, Csdc2, Acox1, Pdk1, Kdm5c, Camsap1, Prnp, Ssh2, Map3k20, Ttc3, Kmt2c, Pbxip1, Ckap5, Gpx4, Fam162a, Abhd17a, Pfdn2, Mkl2, Bod1, Rps27l, Tubb4b, Egflam, Tuba4a, Rapgef2, Dgke, Gas2l1, Cyth3, Plxna2, Erlec1, Abcb1a, Rgs4, Hipk1, She, Plin5, Mcam, Galnt10, Prdm16, Actn4, Mylk, Mgll, Csrp1, Cdc42ep1, Rgs12, Rarg, Sec61g, Eif3j1, Tomm22, Atg101, Pck2, Gja4, Aqp7, Lgals1, Tmem200b, Fgfr3, Esam, Fam213b, Tinagl1, Gpr4, Ccdc32, Tbx2, Slc9a3r2, Nes, Ppp1r14a, Fbxl22, Adamts9, Btnl9, Fabp4, Cd300lg, Egfl7, Slc25a17, Kcnk6, Abcg2, Myl9, Mtmr10, Lrrc8a, Nxpe4, Sox18, Snu13, Ndufaf2, Ajuba, Robo4, Tspan17, Pdgfb, Hmgb1, Ccnd1, Lims2, Psmb5, Zfp697, Sh2d3c, Ndufaf1, Olfr1033, Uchl3, Plekhg2, Vtn, Dach1, Fam57b, Gimap8, Rasl10a, Zmat5, Smox, Mfap1b, Ccdc85a, Tmc6, Pde4c, DHRSX, Enho, Setdb2, Aurkaip1, Il17d, Arl4d, Arhgap27, Inafm2, 3632451O06Rik, Klhl2, Sox17, Ptprn, Tuba1b, Mgmt, Lrrc8c, Gck, Gpihbp1, C1qtnf12, Kcna5, Acta2, Dennd3, Atoh8, Cyb561, Zbtb16, Epha2, Hpcal1, Cd59a, Abi3, Klf10, Rpl41, Sema7a, Cdc42ep4, B4galt4, Mertk, Ttll7, Lfng, Ric3, Gimap1, Sema6b, Lpin3, Tmem88, Notch4, Ucp2, Haus3, Iqsec3, Colq, Adgrg1, Bcl2l1, Angptl4, Slc4a8, Dynlt1c, Mcf2l, Eef1akmt1, Hecw2, Fam117b, C1qtnf2, Scd4, Zkscan3, Eps8l1, Ccdc157, Rab27a, Rbp7, Plaur, Rpl3, Hspb3, Nmb, Ciart, Capza1, 8430408G22Rik, Casc4, Tusc5, 0610040J01Rik, Zfp772, Agap2, Hrct1, Aplp1, Micall2, Tcf15, Slc7a1, Mcpt4, Ltc4s, Gper1, Uchl1, Fam46b, Rtn4rl2, Map3k6, Tubb4a, Nutf2-ps1, Nrg2, Apobec3, Cnga3, Il20rb, Atcay, Ppp1r3e, Efcab7, 2210407C18Rik, Rapgef3, Clca3a1, Kcnmb1, Zfp429, Coro2a, Ang, Rasgef1c, Prps2, Sumo2, Ifi208, Hoga1, Gldc, Sptbn5, Cyp1a1, Sstr3, Cd59b, CN725425, Xlr3b, Fhad1, Xlr4a, Ddx4, Onecut3, Pax6, 4930555G01Rik, Hba-a2, St8sia5, S100a8, Cemip* |
| **C57-IR vs. C57-non-IR Down / (385)** | *Podxl, Gpt2, Ip6k1, Hip1, Adarb1, Plin4, Car8, Map4k3, Usf1, Inafm2, Limk2, Cbx7, Syde1, Zfp740, Wbp1l, Cxcl12, Epb41, Plin5, Trim25, Pald1, Snx33, Pgpep1, 2310011J03Rik, Map4k2, Rgs12, Ndst2, Mlxip, Zfp746, Fam160b1, Tnfsf10, Inpp1, Chd6, Egflam, Palm, Fzd6, Agrn, Tmem18, Cdc42ep1, Zbtb46, Slc35b4, Men1, Uchl3, Lifr, Slc9a3r2, Trim11, Hipk1, Fut11, Sigirr, Cbfa2t3, Klf3, Caskin2, 1110038F14Rik, Gtpbp8, Stard8, Cnnm3, Zfp970, Zfp606, Heg1, Abcc4, Pcdh1, Wdr24, Chst15, Adgrf5, Eml3, Arl14ep, Slc36a1, Max, Mthfsd, Gan, Prodh, Plcg1, Dab2ip, Znhit2, Nbeal2, C130074G19Rik, Pygo2, Lipe, Zfp949, Kdm5d, Golga1, Hyal2, Zfp276, Car4, Fam161b, Zfp398, Smarcd2, Taok2, Nr2f2, Gja4, Tia1, Klhl2, Zfp790, Eln, Papln, Knop1, Cd300lg, Il17rd, Rasal2, Ptprb, Vsir, Hand2, Abcg2, Dach1, Ptgs1, Fn3krp, Dusp7, Oraov1, Trim7, Xpa, Bmp6, Ppcs, Mkl2, Ints5, Rflnb, Npr3, Egfl7, Sh2d3c, Pck2, Xdh, Ttc14, Inf2, Trim12c, Trim21, Zfp729a, Sac3d1, Hspa12b, Lpcat1, Cbx6, Patz1, Ankrd9, Edc3, Slc4a8, Zfp629, Tprn, Tmem88, Rarg, Cd200, Csgalnact1, Bdh1, Commd5, Zhx3, Shprh, Amigo2, St6galnac2, Nav3, Dgke, Zbtb39, Zfp950, Sprtn, Gipc3, Zfp366, Ajuba, Mtmr10, Ston1, Gpihbp1, Ttll7, 9930111J21Rik2, Aqp7, Tmem82, Notch4, Thap7, Zbtb5, Trim2, Zfp952, Ephb4, Zfp526, Klf15, Tbx2, Gstt2, Glce, Zfp780b, Zfp319, Card10, Agap2, Prag1, Zfp958, Ap5b1, Dbndd2, Pik3r1, Mfsd7c, Rrnad1, Btnl9, Plekhf1, Apobec3, Zfp74, Sema7a, 4930578C19Rik, 0610030E20Rik, Ifi44, Ccnj, Adora2a, Slc35c1, Dok4, Osbpl7, Rnf169, Rapgef3, Zfp334, Smim1, Zbtb34, Thsd1, Trim45, Tctn1, Net1, Rasl10a, Trim12a, Zfp84, Def6, Ip6k3, Lims2, Zfp866, Adora1, Spock2, Map3k6, Rnf144b, Snrk, Zcchc3, Zfp646, Sertad3, Hrct1, Klf13, Fbxo46, Gja5, Zfp563, Zfp738, Zfp946, Kif26a, Trim56, Sft2d3, Zfp709, Paqr8, Zfp764, Gata2, Tnfaip8l2, Inip, Tril, Slc45a3, Tmem200b, Lmod1, Zfp35, Tigd5, B3gnt8, Cbx2, Hecw2, Lhx6, Zfp772, Nynrin, Arhgap27, Zfp11, Capn10, Zfp111, Sall2, Nhsl2, Jade2, Zfp809, Alkbh4, Alox12, Rsrp1, Gatsl3, Zbtb6, Zbtb16, Hps6, Zfp113, Zfp101, Mxd4, Zfp870, Tmem229b, Zfp112, Spaar, Vmac, Zfp595, Fam57b, Thrsp, Zfp825, St8sia6, Zfp60, Haus3, Hic1, Kbtbd11, Aplnr, Pdik1l, Smad6, Skida1, Pth1r, Osr1, Syn2, Exoc8, Fzd4, Slx1b, Acer2, Irx1, Spata2l, Homez, Disc1, Tmem267, Sox18, Kcna5, Zfp72, Tbxa2r, Zfp324, Bcl2l1, Arl10, Zfp12, Cngb1, Tmie, Fmo2, Zfp128, Lfng, Zfp189, Ankrd63, Tor4a, Hhipl1, Sptbn5, Zfp235, Lpin3, Nrg2, 2210407C18Rik, Kank3, Zfp953, Neurl1b, Gimap1, Zfp472, Sult1a1, Gdf10, Acot11, Clec1a, Dynlt1c, Irx2, Flt3l, Klhl42, Zfp39, Fam117b, Mmp28, Dclre1b, Itpripl1, Adam33, Zfp748, Wisp2, Tnfrsf25, Atoh8, Ltc4s, Tlr13, Ctu1, Tcf15, D930048N14Rik, Vsig2, Mcf2l, Peg12, Npy1r, 9130019O22Rik, Zfp87, Izumo4, Coro2a, Hif3a, Rnf152, Hbb-bs, Fkbp5, Adh1, Cyp2b10, Trim5, Zfp760, Vps37d, Hba-a1, Pbld1, Zfp763, 4931422A03Rik, Slco1a4, Fam46b, Doc2b, Zfp493, Mycbpap, mt-Nd3, Fam107a, Nrgn, Arrdc2, Lrat, Gabra1, 3830403N18Rik, Tmem240, Pdzd9, Degs2, Slc12a3, Galr2, Ccl28, S100a8, Spag6* |
| **C57-IR vs. C57-non-IR Up / (646)** | *Hspa1b, Adamts4, Plin1, Hspa1a, Hamp, Sln, Il6, Cxcl3, Olr1, Gjb4, Ptx3, Mybphl, Rxfp1, Ifnb1, Cfd, Csf3, Nr4a3, Nptx2, Cyp24a1, Cxcl10, Has1, Osr2, Hist1h4a, Acod1, Il1r2, Cidec, Il17f, Slc6a14, Il17a, Dcstamp, Selp, Sall3, Fgf23, Fam71a, Myl7, Edn2, Sele, Il1a, Tmem163, Dmkn, Krt8, Il1rn, Tnfaip6, Artn, Ccl4, Areg, Ildr1, Klf14, Nts, Serpina3f, Ch25h, Egr4, Dnajb1, Cxcl2, Car3, Hp, Zdbf2, Spic, Clec4e, Rnd1, Ccl3, Tnfsf11, Tnf, Foxj1, Plaur, Myc, Lipg, Hoxa5, Tnfsf18, Irs2, Thbs1, Arid5a, Acp5, Tnfaip3, Mcoln2, Atf3, Plek, Mmp13, Fst, Gadd45g, Ccl5, Rgs1, Arc, Rasgrp1, Krt18, Pdcd1, Pmaip1, Cd83, Slamf7, Hcar2, Gfap, Klf5, Epyc, Tnfsf9, Hmox1, Ereg, Bdkrb2, Socs3, Has2, Tnfrsf10b, Gadd45b, Nlrp3, Gdnf, Fosl2, Maff, Cxcl1, Nr4a2, Stmn4, Ccl2, Nfkbid, Zap70, Nppc, Gng4, Gem, Rgs16, Flrt3, Sphk1, Cxcl5, Bcl3, Zcchc5, Icam1, Hsp90aa1, Lif, Pck1, Frat2, Dusp2, Lilr4b, Pim1, Sbno2, Gdf15, Cmtm5, Ccl7, Gfpt2, Tlr2, Ppp1r15a, Ptgs2, Slc16a3, Fam46c, Casp4, Batf, Csrnp1, Il10, Apold1, Hbegf, Osm, Hsph1, Adamts1, Gpr132, Rasgef1b, Nfil3, Rasl11a, Akap12, Timp1, Fam167a, Il1b, Nfkbiz, Litaf, Marcksl1, Peg10, Stap1, Tgif1, Ifrd1, Ctla2b, Plau, Dnaja1, P2ry10, Ptpn22, Nfkbie, Ikzf4, Lilrb4a, Pde4b, Gprc5a, Phlda1, Cd69, Map3k8, Otud1, Ccno, Gla, Xirp1, Fosl1, Slc16a11, Bmp2, Fam84a, Ccl11, Dusp5, Plekho2, Abra, Ugdh, Tiparp, Gcc1, Sat1, Hspb1, Cbarp, Irf1, Procr, Itgad, Pfkfb3, Fgl2, Bcl2a1d, Slc25a25, Bhlhe40, Socs1, Tmem173, Serpinb8, Nr4a1, Junb, Plk3, Zc3h12a, Egr2, Tmem88b, Csf2rb2, Itpkc, Stx11, Sowahc, Serpine1, Chrna10, Pdpn, Zfp36, Il17ra, Lonrf3, Sertad1, Emp1, Dusp10, Nmrk2, Irf8, Tnc, Neurl3, Cebpd, Tnfrsf12a, Cd180, Samsn1, Trib1, Tnfaip2, Cdkn1a, Zfp948, Bcl6, Ackr2, Egr1, Ctgf, Cdr2, Dusp4, Prr7, Arl5b, Foxf1, Kcne4, B3galt2, Lag3, Ier5, Alkal2, Bdnf, Rcan1, Ier3, Btg1, Runx1, Wsb1, Arid5b, Cass4, Creb5, Bcl6b, Cyr61, Egr3, Timeless, Cfap43, Cldn5, Spty2d1, Rnf122, Gpr183, Nppb, Eif1a, Rel, F2rl1, Adamts8, Ccl24, Actg1, F830016B08Rik, Pde12, Inhba, Sdc4, Pcdh9, Zfand2a, Lrrc32, Tfpi2, Fam26e, Dnajb4, Fos, Btg2, Crem, Ripk3, Rasd1, Tgtp1, Slc15a3, Slc20a1, Rasl11b, Cd274, Rrad, Rnd3, Hcn1, Ccnl1, Mafk, Hilpda, Kif5c, H2-Q6, Rfx2, Arrdc3, Ctla2a, Fosb, Jun, Vcam1, Cited2, Kdm6b, Slc38a2, Birc3, Ier5l, Nupr1, Nedd9, Gas2l3, Cxcr4, Igtp, Loxl4, Relb, B4galt5, Iigp1, Jund, Ifi211, Cd14, Slc10a6, Dusp8, Gja1, Nnmt, Asns, Pxdc1, Ankrd23, Klf6, Pappa, Zbtb10, Plk2, Scml4, Tcf21, Bcr, Hspa2, Odc1, Dusp27, Osgin1, Il4ra, Hdc, Ripk1, Nov, Ifi207, Lcp2, Slc16a13, Kctd11, Cd24a, Sema4c, Fbxo30, Ngf, Serpinh1, Usp27x, Lmna, Utp14b, Itga5, Prdm1, Spin4, Rpl17, Sgms2, Asb4, Zswim4, Errfi1, Siah2, Ddit3, Fam46a, Fam107b, Cxcl9, Atf4, Pnrc1, Rlim, Hspa1l, Srxn1, H3f3b, Gadd45a, Actb, Pramef8, Trim16, Uap1, Slc2a1, Zfp36l1, Col5a3, Bambi, Isg15, H2-Q4, Coq10b, Plat, Slc5a3, Sox9, Tifa, Lhfpl2, Cnnm4, Azin1, Klhl40, Ptp4a1, Grrp1, Lrrn4cl, Bag3, Ier2, Hspa8, Sirt1, Pgf, Lox, Endou, Kctd12, H2-Q7, Myd88, Rhoc, Slc7a5, Efs, Tra2b, Nfkb2, Pgm1, Itgb8, Spry4, Fzd5, Ndnf, Nop58, Ptger4, Rassf1, Arl13b, Mapk6, Polr2a, Mxd1, Cd44, Srp54a, Wt1, Emd, Rnf19b, Rabgef1, Mest, Spry2, Arrdc4, Pvr, C5ar1, Rap1b, Gnai3, Lmcd1, Abi3bp, Dnaja4, Stat3, Cd55, Tsc22d2, Rab7b, Ifi204, Bach1, Btg3, Eva1a, Arl4c, Hspa5, Mmp14, AW011738, Elf1, Tubb6, Pi4k2b, Rela, Cbx4, C3ar1, Retreg1, Lima1, Frmd6, Bcar1, Tlr4, Ralgds, 1190002N15Rik, Ackr3, Sde2, Taf7, Irgm1, Rab20, Map3k14, 1810055G02Rik, Zbtb21, Upp1, Cep290, Col15a1, Flnc, Cwc25, Gbp2, Ldlr, Vwa1, Ccrl2, Sik1, Edn1, Zbtb2, Rpl22l1, Pcna, Bcl10, Zfand5, Ubc, Bmp2k, Mcl1, Ippk, Synpo2l, Impact, P4ha1, Fam43a, Hs3st1, Ednrb, Cxcl14, Clk1, Ptpn12, Hmgcr, Nes, Anxa1, Brd2, H2-K1, 3110082I17Rik, Ppp1r2, Ankrd1, Manf, Dnajb9, Tpm3, Osmr, Insig1, Enah, Epha2, B4galt3, 1110002E22Rik, Slc3a2, Hif1a, Impdh2, Sash1, Tppp3, Twf2, Icosl, Grasp, Ret, Prkab1, Arf4, Tbx20, Filip1l, Zfp326, Ppargc1b, F2r, Klhl41, Dedd2, Cyp51, Thbd, Midn, Vps37b, Jmjd1c, Tuba1c, Ptma, Srgn, Sdcbp, Pde3a, Ggct, Morf4l2, Med7, Noct, Pdlim1, Actr3, Myl1, Fbn1, Nfatc1, Eif4e, Txndc11, Col5a1, Lmod2, Tm4sf1, Lama4, Gata4, Zyx, Clic4, Rhbdf2, P2ry2, Adnp2, Ddx3x, Calr, Zbtb11, Peli1, Pspc1, Fndc1, Rbm24, Mid1ip1, Ccnt1, Ythdc1, Pqlc1, Lysmd3, Txnrd1, Nasp, Gclc, Ascc3, Nup98, Il13ra1, Hmgcs1, Ndel1, Zcchc6, Csf1, Ipmk, Lamb1, Hipk3, Fbxw7, Stip1, Ahsa2, Dpysl3, Hist1h1c, Rps6ka3, Cpeb4, Srf, Zfp703, Slmap, Top1, Homer1, Rsrc2, Gna13, Hsp90ab1, Tnxb, Katna1, Ywhaz, Rbm18, Gls, Cnot4, Smarca5, Hyou1, Bcor, Carnmt1, Golph3, Ets2, Lims1, Vasp, Mapre1, Cdc42ep3, Slain2, Kras, Slc25a36, Pgm5, Hspd1, Lamc1, Ccar1, Ptpn21, Hopx, Fasn, Arih1, Rhoa, Rgl1, Rpl23a, Rpl3, Ube2d3, mt-Co1, Rtn4* |
| **Slit2-IR vs. C57-IR Down / (112)** | *Aurkaip1, Cox7a1, Cnot6l, Klhl9, Rpl30, Cep83, Dnajc25, Smim4, mt-Co1, Ss18l2, Ublcp1, Eif2ak1, Ccm2l, mt-Nd2, Wdr77, Tnfsf10, St3gal5, Dusp18, mt-Nd1, Tomm22, Chp1, Eef1akmt1, Gbp9, Pde7a, N4bp2l2, Etfbkmt, Pcp4l1, Cd59a, Ednra, Prmt9, Ubc, Skap2, Acaca, Haus2, 1110034G24Rik, Zfp874b, Ppp1r3c, C2cd2, mt-Nd3, mt-Atp6, Sfi1, Amy1, Tnni3k, Ndufaf1, Gbp5, Rgs7bp, Mt1, Zmat5, Hjurp, Acot1, Adgre1, Frmd5, Tmem67, Rpl3, Prps2, Ccdc32, Cd274, Lrtm1, Slc25a17, H2-Q6, Dnajc17, Apobec3, Cd84, Nutf2-ps1, Ppargc1a, H2-Q7, Vwa3a, Ccl24, Srp54a, Irgm2, Zfp697, Gbp4, Uchl1, Capza1, Serpina3n, Mall, Cd59b, Itgam, Ccl6, Igtp, Zkscan3, Ccdc112, A930033H14Rik, Tmem88b, Ms4a6d, Hmgb1, Grin2c, Bnc2, 0610040J01Rik, Ifitm1, Mfap1b, Lrrc4c, Sstr3, Slamf7, Trpm2, Rasgrp1, Clca3a1, P2ry10, Crb1, A130051J06Rik, Lcn2, Hoxa1, Ager, Slc7a1, Cxcl13, Xlr4a, Il5, Zg16, Hba-a2, Sftpa1, Scgb1a1, Sftpc* |
| **Slit2-IR vs. C57-IR Up / (183)** | *Ccnb1ip1, Gdf7, Dpp6, Pcna-ps2, Oit3, Gabra1, Mfsd13b, Atp1a4, Ifi202b, Plppr5, Tnmd, Apol8, Cntn4, Pitx2, Nfasc, C1s2, Gabrb2, Cpne5, Bex1, Pvalb, Akap2, Ryr1, Eif3j2, Vwc2, Fer1l6, Syndig1, Dlgap1, Baiap2l2, Mgat5b, Slc7a11, Gcgr, Rab3b, Col11a1, Zfp969, Ckmt1, Edn2, 1810041L15Rik, C1qtnf6, Drd2, Pappa2, Casq1, Glcci1, Shank2, Tceal5, C530008M17Rik, Elfn2, Fgf10, Pcdhb13, D430019H16Rik, Slc51a, Wdfy1, Helt, Fam84a, Flrt1, Rnf17, Stab2, Sema6c, Nos1, Kif1a, Ripk4, Ankrd2, Npas2, Shisa2, Padi2, Shisa4, Cdkn2b, Rab4a, Rab15, Col8a2, Srgap3, Armcx4, Sfrp2, Kcna1, Frem2, Irx2, Col12a1, Myh7, Chd5, Cryba4, Cacna1g, Foxo6, Proser3, Crocc, Kcne1, Alad, Hdhd3, Sorl1, Gchfr, Wisp1, Ucp3, Olfml2b, Robo2, Irx5, Gdf10, Ankrd1, Gatm, Fstl3, Lad1, Abca4, Aldh1l2, Sept1, Ddr1, Capn3, Obsl1, Nrxn1, Loxl2, Disc1, B9d1, Neurl1a, Col15a1, Ift122, Col1a1, Pdgfrl, Sh3rf2, Slc2a10, Trim7, Col4a3, Pcdh17, Stc2, Lgi1, Gnb3, Hr, Prelp, Irs1, Itgb6, Asap3, Hspa1a, A930018M24Rik, Pak4, Ramp1, Dapk2, Lpin3, Epn3, Igsf1, Trip6, Camk2a, Plxnb1, Tmem150c, Col6a6, Lrfn4, Kcnq4, Col6a2, Nt5c1a, Irf2bpl, Acot11, Ppp1r10, Scn4b, Pla2g6, Ptpre, Trp53, Cux2, Arvcf, Rftn1, Gsg1l, Kcna7, Igdcc4, Fam20c, Btbd9, Kctd17, Mapt, Hcn2, Col5a1, Wisp2, Pcdhgc3, Dvl1, Phf5a, St6galnac4, Nol12, Tcf3, Itga9, Fubp3, Irf2bp2, Prnp, Slc16a2, Ascc2, Hspg2, Fads3, Taf6, Ddx18, Tsc22d4, Gnas, Trim8, Lama2* |
| **C57-IR vs. C57-non-IR Down + Slit2-non-IR vs. C57-non-IR All / (60)** | *Rasl10a, Dynlt1c, Tbx2, Hecw2, Ttll7, Hipk1, Notch4, S100a8, Ajuba, Zbtb16, Tmem200b, Haus3, Pck2, Egfl7, Dgke, Sox18, Rgs12, Agap2, Fam117b, Hrct1, Inafm2, Gpihbp1, Klhl2, Coro2a, Sema7a, Rarg, Lfng, Mkl2, Map3k6, Btnl9, Cdc42ep1, Zfp772, Slc4a8, Egflam, Lims2, Aqp7, Tcf15, Plin5, 2210407C18Rik, Uchl3, Mtmr10, Nrg2, Dach1, Slc9a3r2, Tmem88, Abcg2, Rapgef3, Fam46b, Sh2d3c, Bcl2l1, Cd300lg, Gimap1, Sptbn5, Atoh8, Mcf2l, Kcna5, Gja4, Ltc4s, Arhgap27, Fam57b* |
| **Slit2-IR vs. C57-IR Up + Slit2-non-IR vs. C57-non-IR All / (36)** | *Bex1, Zfp969, Ifi202b, Wdfy1, Ripk4, Olfml2b, Lama2, Eif3j2, Sorl1, Elfn2, A930018M24Rik, Tmem150c, 1810041L15Rik, Irs1, Prnp, Gchfr, Stab2, Fer1l6, Aldh1l2, Sh3rf2, Dvl1, Col11a1, Gatm, Camk2a, Armcx4, Glcci1, Stc2, Slc7a11, C1qtnf6, Vwc2, Pcna-ps2, Asap3, Akap2, Ascc2, Helt, Rab4a* |
| **C57-IR vs. C57-non-IR Up + Slit2-non-IR vs. C57-non-IR All / (27)** | *Hcn1, Nes, Sox9, Ret, Abi3bp, Fst, Pck1, Lilr4b, Ppargc1b, 1110002E22Rik, Cidec, Kif5c, Cfd, Gata4, Tbx20, Hamp, Plin1, Rpl22l1, Bcl6, Plaur, Rgs1, Hp, Fbxw7, Gja1, Cxcl14, Epha2, Xirp1* |
| **Slit2-IR vs. C57-IR Down + Slit2-non-IR vs. C57-non-IR All / (23)** | *Mfap1b, Uchl1, Cd59a, Cd59b, Xlr4a, Hba-a2, Hmgb1, Slc7a1, 0610040J01Rik, Aurkaip1, Zfp697, Zkscan3, Nutf2-ps1, Zmat5, Sstr3, Tomm22, Prps2, Eef1akmt1, Clca3a1, Capza1, Ccdc32, Ndufaf1, Slc25a17* |
| **Slit2-IR vs. C57-IR Down + C57-IR vs. C57-non-IR Up / (12)** | *mt-Co1, P2ry10, Tmem88b, Igtp, Rasgrp1, Cd274, Slamf7, Srp54a, Ubc, H2-Q6, Ccl24, H2-Q7* |
| **Slit2-IR vs. C57-IR Up + C57-IR vs. C57-non-IR Down / (6)** | *Gdf10, Disc1, Irx2, Acot11, Trim7, Wisp2* |
| **Slit2-IR vs. C57-IR Up + C57-IR vs. C57-non-IR Up / (6)** | *Col15a1, Fam84a, Edn2, Ankrd1, Col5a1, Hspa1a* |
| **Slit2-IR vs. C57-IR Up + C57-IR vs. C57-non-IR Down + Slit2-non-IR vs. C57-non-IR All / (2)** | *Gabra1, Lpin3* |
| **Slit2-IR vs. C57-IR Down + C57-IR vs. C57-non-IR Down / (2)** | *Tnfsf10, mt-Nd3* |
| **Slit2-IR vs. C57-IR Down + C57-IR vs. C57-non-IR Down + Slit2-non-IR vs. C57-non-IR All / (1)** | *Apobec3* |
| **Slit2-IR vs. C57-IR Down + C57-IR vs. C57-non-IR Up + Slit2-non-IR vs. C57-non-IR All / (1)** | *Rpl3* |
